# HIF-1 attenuates high-fiber diet-mediated proliferation and stemness of colonic epithelium

**DOI:** 10.1080/19490976.2025.2543123

**Published:** 2025-08-19

**Authors:** Pollyana Ribeiro Castro, Renan Oliveira Corrêa, Monara Kaélle Sérvulo Cruz Angelim, Vinícius de Rezende Rodovalho, Mariane Font Fernandes, Vinícius Dias Nirello, Arilson Bernardo dos Santos Pereira Gomes, Jaqueline de Souza Felipe, Lais Passarielo Pral, Sarah de Oliveira, Patrícia Brito Rodrigues, José Luís Fachi, Guilherme Reis-de-Oliveira, Bradley J. Smith, Victor C. Carregari, Nicolas G. Shealy, Catherine Shelton, Daniel Martins-de-Souza, Pedro M. Moraes–Vieira, Mariana Xavier Byndloss, Patrick Varga-Weisz, Marco Aurélio Ramirez Vinolo

**Affiliations:** a Laboratory of Immunoinflammation, Department of Genetics, Evolution, Microbiology and Immunology, Institute of Biology, University of Campinas, Campinas, SP, Brazil; b Université Paris Cité, Imagine Institute, INSERM UMR1163, Laboratory of Intestinal Immunity, Paris, France; c Laboratory of Immunometabolism, Department of Genetics, Evolution, Microbiology and Immunology, Institute of Biology, University of Campinas, Campinas, SP, Brazil; d Bioinformatics Laboratory (LABINFO), National Laboratory for Scientific Computing (LNCC), Petrópolis, Brazil; e Department of Pathology and Immunology, Washington University School of Medicine in Saint Louis, Saint Louis, MO, USA; f Laboratory of Neuroproteomics, Department of Biochemistry and Tissue Biology, Institute of Biology, University of Campinas (UNICAMP), Campinas, SP, Brazil; g Boldrini Research Center, Campinas, SP, Brazil; h Department of Pathology, Microbiology & Immunology, Vanderbilt University Medical Center, Nashville, TN, USA; i Experimental Medicine Research Cluster (EMRC), Campinas, SP, Brazil; j National Institute of Science and Technology in Modeling Human Complex Diseases with 3D Platforms (INCT Model3D), São Paulo, São Paulo, Brazil; k Obesity and Comorbidities Research Center (OCRC), University of Campinas, Campinas, SP, Brazil; l Howard Hughes Medical Institute, Vanderbilt University Medical Center, Nashville, TN, USA; m International Laboratory for Microbiome Host Epigenetics, Department of Genetics, Evolution, Microbiology, and Immunology, Institute of Biology, University of Campinas, Campinas, SP, Brazil; n School of Life Sciences, University of Essex, Colchester, UK

**Keywords:** Hypoxia, dietary fiber, inulin, metabolism, microbiota, intestinal stem cells

## Abstract

The complex interplay between diet, microbiota, and host is exemplified by the effects of dietary fiber on the intestine. Inulin ingestion triggers epithelial changes in the colon that depend on microbiota-derived molecules, including enhanced proliferation, increased mucus production, and elevated antimicrobial peptide secretion. Here we employed a multilayered and multi-omics approach, including dietary interventions, intestinal organoids, and both genetic and pharmacological interventions to investigate the impact of inulin on two aspects of diet-microbiota-host interactions: intestinal hypoxia and hypoxia-inducible factor (HIF)-1 signaling in intestinal epithelial cells (IECs). We found that inulin, a soluble fiber, promotes intestinal hypoxia, stabilizing HIF-1 in IECs in a microbiota- and host-dependent manner. Furthermore, we show that HIF-1 stabilization modulates intestinal stem cell (ISC) function through metabolic reprogramming in a microbiota-dependent manner. Our findings reveal an unrecognized role for HIF-1 in orchestrating microbiota-dependent epithelial metabolism and proliferation in the colon, underscoring the complexity of diet-microbiota-host interactions.

## 1. Introduction

The gut epithelium consists of a single layer of cells derived from intestinal stem cells (ISCs) located at the base of the intestinal crypts.^1^ Beyond its absorptive and secretory roles, the epithelium serves as a physical barrier, protecting the lamina propria from potentially harmful components in the intestinal lumen. To maintain host homeostasis, this dynamic structure continuously adapts to environmental factors such as microbiota composition, dietary intake, and oxygen levels.^2,3^ Understanding how these factors communicate with each other in a steady state is important and our study seeks to bring more clarity to these complex interactions.

A healthy microbiota features high taxonomic diversity, microbial gene richness, and a stable core, essential for epithelial functions.^4^ The microbiota maintains barrier integrity, facilitates nutrient metabolism, regulates immune responses, promotes cellular regeneration, and provides colonization resistance, collectively fostering intestinal health and preventing intestinal diseases.^5^ Intestinal epithelial cells (IECs), in turn, express pattern recognition receptors (PRRs), such as Toll-like receptors (TLRs), which detect microbial components and mediate responses to both beneficial and harmful microbes.^6^ Furthermore, IECs sense metabolites derived from microbial breakdown of dietary components, such as short-chain fatty acids (SCFAs) or aryl hydrocarbon receptor (AhR) ligands including tryptophan derivatives like kynurenine, indole-3-acetic acid, and indole-3-propionic acid. These metabolites play critical roles in energy metabolism, production of mucus and antimicrobial peptides, and mucosal healing.^7–9^

Additionally, dietary choices greatly influence the intestinal environment. Consumption of prebiotics, such as dietary fiber inulin or fructooligosaccharides, offers health benefits by modulating specific bacterial strains and boosting the production of microbial-derived metabolites.^10^ Recent findings reveal that inulin intake remodels the colonic epithelium by enhancing ISC proliferation and differentiation, a process dependent on coordinated interactions between the gut microbiota and the immune system.^11^ In addition to fibers, several other dietary signals have been shown to significantly influence ISC fate decisions,^12^ highlighting the pivotal role of diet in shaping epithelial renewal, composition and functions.

Fluctuations in intestinal oxygen levels, shaped by local metabolic activity and physiological conditions, impact the microbiota and epithelial homeostasis.^13,14^ Oxygen levels in the colonic epithelium decline from ~ 3% in the submucosa to < 1% in the luminal surface.^15^ The SCFA butyrate sustains hypoxia by promoting oxidative metabolism in IECs,^16^ consuming oxygen, and stabilizing hypoxia-inducible factor 1 (HIF-1), which in turn regulates glucose use and mitochondrial biogenesis, supporting epithelial homeostasis under hypoxic conditions.^17,18^

Intestinal hypoxia and HIF-1 activation by the gut microbiota are essential for maintaining barrier integrity,^19,20^ with disruptions driving pathological conditions. For instance, reduced butyrate-producing *Clostridia* after antibiotic treatment increase epithelial oxygenation, allowing expansion of pathogenic, aerobic *Salmonella*.^21^ Similarly, high-fat diets impair IECs mitochondrial oxygen uptake, increasing susceptibility to anaerobic pathobionts like *E. coli*.^22^ Hypoxia also worsens intestinal inflammation and epithelial damage during *Salmonella enterica* serovar Typhimurium infection,^23^ emphasizing the need to understand how hypoxia mediates beneficial or harmful responses.

In this study, we explored how dietary fiber, microbiota, and hypoxia influence epithelial responses. Our findings revealed that both microbiota- and host-mediated mechanisms contribute to increased colonic hypoxia and HIF-1 activation in intestinal epithelial cells after inulin ingestion. Using conditional knockout mice, we identified HIF-1α as a key regulator of cell proliferation and intestinal stem cell function in response to high-fiber diet. We find these effects are associated with metabolic regulation of epithelial cells. Together, our findings reveal the intricate role of dietary and microbiota-derived factors in shaping oxygen availability and intestinal epithelium function.

## 2. Materials and Methods

### 2.1. Mice

All animal procedures described in this project were approved by the Ethic Committee on the Use of Animals (CEUA) of the University of Campinas (Protocols #4537–1/2017, #5881–1/2021, #5967–1/2024) and Vanderbilt University Medical Center. C57BL/6 mice were purchased from the Multidisciplinary Center for Biological Research (CEMIB – IB/UNICAMP). ODD-luciferase, HIF-1α^fl/fl^, Vhl^fl/fl^, Pparg^fl/fl^, Lgr5-EGFP-IRES-creERT2 and Villin-cre mice were purchased from Jackson Laboratory. Conditional knockout mice (HIF-1α^ΔIEC^, VHL^ΔIEC^ and Pparg^ΔIEC^) were generated after crossing mice with the floxed genes (HIF-1α^fl/fl^, VHL^fl/fl^ and Pparg^fl/fl^) and mice that express the Cre recombinase enzyme under the control of the Villin promoter (Vil-Cre) or Lgr5 (Lgr5-EGFP-IRES-creERT2, Lgr5-Cre). HIF-1α^ΔISC^ mice were generated after crossing Lgr5-Cre with Ai14 (B6.Cg-Gt(ROSA)26Sortm14(CAG-tdTomato)Hze/J) and HIF-1α^fl/fl^. Other strains were purchased from Jackson Laboratory or provided by collaborators from the Department of Microbiology, Institute of Biological Science of the Federal University of Minas Gerais. All mice were kept in the Animal Facility of the Department of Genetics, Evolution, Microbiology and Immunology of the Institute of Biology of the University of Campinas in cages with a filter on the lid and had *ad libitum* access to water and sterilized food. C57Bl/6N germ-free mice (Taconic, B6NTac – GF) were bred in-house and moved into a positive pressure cage system at 3 weeks old in the Animal Facility at Vanderbilt University Medical Center. Adult male and female mice aged 8 to 12 weeks were used in all experiments.

### 2.2. Dietary approaches

Control and inulin diets were prepared at the Laboratory of Cereals, Roots, and Tubers (FEA/UNICAMP) according to the American Institute of Nutrition recommendations (AIN93).^24^ The control diet was composed of 5% insoluble fiber cellulose, and the inulin-enriched diet was composed of 5% cellulose + 10% inulin (soluble fiber). Details of each diet composition can be found in our previous publication.^11^ Mice were fed for 3–4 weeks, unless otherwise mentioned. Body weight was measured for each animal before and after the diets. After euthanasia, intestines were harvested and measured, with their values normalized by each individual animal’s body weight. For antibiotic experiments, C57BL/6 mice received a mix of 1 g/L ampicillin, 1 g/L neomycin, 1 g/L metronidazole, and 0.5 g/L vancomycin^25^ in drinking water for 30 days, while being fed with the inulin-enriched diet. For etomoxir intervention, mice on the inulin diet were treated with etomoxir (20 mg/kg of body weight in 100 µL) or vehicle (100 µL of PBS) intraperitoneally, 24 hours and 2 hours before euthanasia.

### 2.3. Analysis of bacteria load

To confirm microbiota depletion, fecal samples were collected from mice before and after diet and antibiotic treatments to assess bacterial load. Briefly, 50 mg of fecal material was used for genomic DNA extraction using the PureLink Microbiome DNA Purification Kit (Thermo Fisher Scientific). Bacterial load was quantified by quantitative real-time PCR (qPCR) using primers targeting a conserved region of the Eubacterial 16S rDNA gene (E338F forward: 5′-ACTCCTACGGGAGGCAGCAGT-3′ and U1407R reverse: 5′-ATTACCGCGGCGGCTGCTGGC-3′), as previously described by Durand et al. (2010).^26^ A standard curve was established using serial dilutions of *Escherichia coli* genomic DNA.

### 2.4. Gnotobiotic mice

Germ-free mice were monocolonized with individual bacterial strains via oral gavage using an inoculum of 1 × 10^9^ CFU in 100 µL. Briefly, *Bacteroides ovatus* (ATCC 8483) and *Bacteroides thetaiotaomicron* (ATCC 29148D) strains were cultured anaerobically at 37°C in Brain Heart Infusion supplemented (BHIS) broth or on BHIS agar plates. For the inoculum preparation, bacteria were streaked onto BHIS agar plates. Single colonies were used to start cultures in BHIS broth. Bacteria were harvested by centrifugation at 3,000 ×g for 8 min and then pellets were resuspended in 1 mL fresh media. The optical density (OD) at 600 nm (OD600) was measured, and the number of bacteria was adjusted to 1 × 10^10^ CFU/mL. A 0.1 mL suspension was then administered to each mouse. Fecal pellets were collected weekly under sterile conditions, weighed, and immediately homogenized in 1 mL of PBS. Serial dilutions were prepared and plated on BHIS agar to quantify bacterial colonization by colony counting.

### 2.5. Analysis of luciferase activity

HIF-1 reporter mice (ODD-luciferase) were fed a control or inulin-enriched diet for 30 days. In accordance with a published protocol,^27^ mice were anesthetized with isoflurane followed by intraperitoneal injection of luciferin (50 mg/kg). Images were taken every min for 5 min on the IVIS Spectrum In Vivo Imaging System. Representative images shown in this manuscript were taken 5 min post luciferin inoculation. After euthanasia, the intestine of these mice was harvested, and a 1.5 cm of the proximal portion of the small intestine and colon was collected for the detection of luciferase activity *ex vivo*. To do so, tissues were first processed with 125 μL of lysis buffer +100 μM cobalt chloride using a tissue homogenizer. Next, samples were snap-frozen in liquid nitrogen and thawed at 37°C for 1 min each, three times. After centrifugation (16,000 ×g, 15 sec, 4 °C), the supernatant was collected, and luminescence (590/35) was measured (Synergy HT Biotek) immediately after the addition of the substrate. Results were normalized to total protein content, as determined by the Bradford assay.

### 2.6. Crypts isolation and single cell dissociation

Colons were harvested, opened longitudinally, washed in cold PBS to remove feces. They were then cut into small pieces and placed in microtubes with Hanks’ balanced salt solution (HBSS) containing 10 mM EDTA and 1% P/S (penicillin/streptomycin). The samples were incubated with agitation (1000 rpm, 37 °C) for 20 min, after which the tissue pieces were removed, and the tubes were centrifuged for 5 min (300×g, 4°C). The pellet was washed with cold HBSS, centrifuged again, and resuspended in HBSS supplemented with 0.04% BSA, 1% P/S, and 10 mM dithiothreitol (DTT) to remove the mucus. After 10 min of incubation at room temperature, cold PBS supplemented with 0.04% BSA was added, and the suspension was filtered through a 70-μm cell strainer.

### 2.7. Histological analysis and hypoxyprobe

Colon samples were fixed in 4% formalin followed by paraffin embedding. Tissue slides were obtained and stained with Hematoxylin – Eosin (H&E) for histological analysis. Hypoxia detection was performed using the Hypoxyprobe™-1 kit (Hypoxyprobe, Inc.). Briefly, mice were injected intraperitoneally with pimonidazole solution (PMDZ) (60 mg/kg of body weight) 90 min before euthanasia. Colon tissues were fixed in 10% buffered formalin, paraffin-embedded, and incubated with monoclonal rabbit anti-pimonidazole IgG1 (Mab 4.3.11.3). Slides were washed and then incubated with a secondary antibody (Cy5-conjugated goat anti-rabbit antibody from Jackson ImmunoResearch Laboratories). The slides were mounted using SlowFade Gold Mountant with DAPI (Molecular Probes). Hypoxia scoring was performed based on the degree of epithelial staining: 0 (no hypoxia), 1 (mild focal hypoxia), 2 (moderate multifocal hypoxia), and 3 (intense diffuse hypoxia), as previously described.^28^ Images were acquired using Cytation 9 (Synergy HT Biotek) and analyzed with ImageJ software.^29^

### 2.8. EdU staining

5-ethynyl-2´-deoxyuridine (EdU) was prepared at 10 mM in PBS and 100 µL was injected i.p. per animal 90 min before euthanasia. Colon tissues were fixed in 4% formalin followed by paraffin embedding. The slides were labeled with Click-It Plus EdU Kit (ThermoFisher Scientific) for incorporation of fluorescent azide (AlexaFluor) followed by counterstaining with DAPI using SlowFade Gold Mountant (Molecular Probes). Images were obtained using Cytation9 equipment (Synergy HT Biotek) and analyzed using ImageJ software. For *in vitro* experiments, organoids were incubated with 100 µM of EdU for 90 min (5% CO_2_ and 37 °C). After this, the medium was removed, and the organoids were collected and labeled with Click-It Plus EdU Kit for flow cytometry analysis.

### 2.9. Intestinal organoids

The distal portion of the colon was collected, washed in cold PBS, and incubated in PBS +10 mM EDTA for 30 min, at 37°C, shaking at 1000 rpm. After PBS washes, isolated crypts were counted and then seeded in Matrigel™ (Corning). Culture medium contained: Advanced DMEM (Gibco), Noggin (200 ng/mL; Peprotech), R-spondin (500 ng/mL; R&D or Sino Biological), epidermal growth factor (EGF; 40 ng/mL; R&D), N-acetyl-L-cysteine (1 μM; Sigma-Aldrich), Chiron (10 μM; Sigma-Aldrich), B27 (1X; Life Technologies), and Y-27632 dihydrochloride monohydrate (20 ng/mL; Sigma-Aldrich). Culture was maintained at 37°C in fully humidified chambers containing 5% CO_2_. Clonogenicity and organoid volume were determined 5 days after seeding. Etomoxir (3 or 12.5 µM), oligomycin (1 µM), antimycin (1 µM), rotenone (100 nM) or 2-deoxyglucose (2-DG; 50 mM) were added to the organoids culture 24 h after initial plating. EdU (100 µM) was added to the organoid cultures 90 min prior to harvesting to assess cell proliferation by flow cytometry. To collect the organoids, the medium was removed, and 1 mL of ice-cold PBS was added to the wells containing the organoids. After this, the Matrigel® was broken with the aid of a pipette tip and the supernatant was collected and centrifuged (300×g, 5 min, 4°C). The resulting pellet was used in different analyses. Detection of EdU-labeled cells was performed using the Click-iT™ Plus EdU Flow Cytometry Kit (ThermoFisher Scientific).

### 2.10. MitoTracker green assay

To evaluate the mitochondrial mass of organoids, we used the probe MitoTracker green (Invitrogen). For this, organoids were incubated with 50 nM of this probe for 30 min, then the medium was removed, and the organoids were washed with 1 mL of cold PBS. After this, we proceed with cell viability and EdU labeling. Cells were then analyzed on a flow cytometer, as described below.

### 2.11. Flow cytometry

Intestinal epithelial cells from colon and organoids cells were obtained as previously described by Corrêa and cols (2023)^11^. To exclude non-viable cells, a live/dead viability assay (Zombie Aqua^TM^ Fixable Viability Kit, Biolegend) was used. Intestinal epithelial cells were gated in live/dead negative population and EdU and/or MitoTracker positive cells (FITC positive) to evaluate proliferation and mitochondrial mass. All cells were diluted in Fluorescence-Activated Cell Sorting (FACS) buffer. Staining of the live/dead and surface antigens was performed in the dark, at 4°C, for 20 min. HIF-1α and Ki67 were stained with monoclonal antibodies after fixation and permeabilization of isolated cells. Samples were analyzed using a FACSymphony™ (BD Biosciences) and FACSDiva™ Software (BD biosciences). All FACS data were analyzed using FlowJo software (v10.1; Becton Dickinson).

### 2.12. Cell sorting

Reporter mice Lgr5-Cre; Ai14; HIF-1α^fl/fl^ with specific deletion of HIF-1α in ISCs (HIF-1α^ΔISC^) and their controls Lgr5-Cre; Ai14; HIF-1α^fl/-^ (HIF-1α^fl/-^) were fed with inulin diet for 3 weeks and received a single tamoxifen (80 mg/kg) injection i.p. 24 hours before the euthanasia. Intestinal epithelial cells from colon and organoids cells were obtained as previously described. To exclude non-viable cells, a live/dead viability assay (Zombie Aqua^TM^ Fixable Viability Kit, Biolegend) was used. IECs were gated in live/dead negative population and tdTomato positive cells. Cells were sorted using FACSDiscover^TM^ S8 (BD Biosciences) and collected in culture media for organoids. FACS-sorted Tomato+ cells were plated for organoids culture as previously described.

### 2.13. Seahorse assay

For real-time analysis of OCR (Oxygen Consumption Rate), colon organoids were analyzed with an XFe96 Extracellular Flux Analyzer (Seahorse Bioscience) according to manufacturer’s instructions and as described by Ludikhuize et al. (2021).^30^ Briefly, 20 organoids in 3 µL of Matrigel® per well were seeded onto an XF96 plate (Seahorse). Due to differences in the growth rates between HIF-1α^ΔIEC^ and HIF-1α^fl/fl^ organoids, the HIF-1α^fl/fl^ organoids were plated two days earlier than the HIF-1α^ΔIEC^ organoids and subsequently grown in complete organoid culture media for 4 days prior to the analysis. This approach ensured that, at the time of measurement, organoids from both groups were of comparable size. Three or more consecutive measurements were obtained under baseline conditions and after the sequential addition of specific inhibitors/activators from the metabolic tests. For the mitochondrial stress test, we added 1 µM of oligomycin, 2 µM of FCCP (fluoro-carbonyl cyanide phenylhydrazone), and 100 mM of rotenone +1 µM of antimycin A (all Sigma-Aldrich reagents). The results of the Seahorse measurements were normalized based on the number of organoids present in the culture.

### 2.14. Single-cell RNA sequencing data analysis

Single-cell RNA sequencing (scRNA-seq) data was obtained from our previous study.^11^ All cell annotations and dimensionality reductions used in this analysis were retained, as described in the original publication. Differentially expressed genes (DEGs) were identified using the FindMarkers function from the Seurat package (version 5.1.0). This function employs the Wilcoxon Rank-Sum test to compare two groups of cells. Default parameters were used unless stated otherwise, and p-values were adjusted for multiple testing using the Bonferroni correction method. Gene ontology analysis was performed using DEGs. The R package enrichR was employed for Gene Ontology (GO) function annotation and Kyoto Encyclopedia of Genes and Genomes pathway (KEGG) enrichment analysis. Enriched status was determined based on a false discovery rate (FDR) < 0.05. Only GO terms with 2 to 1000 assigned genes were considered for enrichment status. Redundancy reduction analysis was performed using the rrvgo package in R, where the calculate SimMatrix() function estimated semantic similarities between GO terms via the Wang method, and terms with a similarity threshold higher than 0.5 were grouped by the reduceSimMatrix() function. The smallest adjusted P-value was assigned for different GO terms grouped under the same parental GO term, and duplicates were removed. Only the top 5 terms related to intestine were included in the graphs. To verify the overlap of expressed genes between the cells, Venn diagrams were generated using the ggvenn package (https://github.com/yanlinlin82/ggvenn.). Expression levels of oxidative phosphorylation (OXPHOS)-related genes, previously identified through gene ontology analysis, were averaged using Seurat by cell type, treatment, and sample ID. Heatmaps were generated with the dittoSeq package, displaying Z-score normalized expression across intestinal stem cells, transit-amplifying cells, and enterocytes.

### 2.15. Quantitative gene expression

Total RNA was extracted from colonic epithelial cells using the PureLink™ RNA Mini Kit (Thermo Fisher Scientific), and its concentration and purity were assessed by NanoDrop spectrophotometer. cDNA was synthesized from 1 µg of RNA using the High-Capacity cDNA Reverse Transcription Kit (Applied Biosystems). RT-qPCR was performed using Power SYBR™ Green PCR Master Mix (Applied Biosystems) on a CFX384 Touch Real-Time PCR System (Bio-Rad). Reactions were conducted in triplicate in a 10 µL volume, with gene-specific primers (Table 1). The thermal protocol consisted of 95°C for 10 min, followed by 40 cycles of 95°C for 15 s and 60°C for 1 min. Specificity was verified by melting curve analysis. Gene expression was normalized to β2-microglobulin as the housekeeping gene, and relative expression levels were calculated using the 2^−ΔΔCt method. Data analysis was performed on CFX Manager software (Bio-Rad) with β2-microglobulin (*B2m*) serving as the reference gene due to its stability and invariant expression across groups.

**Table 1.** Primer sequences used for RT-qPCR analysis.

Gene	Primer direction	Sequence (5‘→ 3’)
***Hif1a***	Forward	ATCTCGGCGAAGCAAAGAGTC
	Reverse	TGGGGAAGTGGCAACTGAT
***Slc2a1***	Forward	CTTTGTGGCCTTCTTTGAAGT
	Reverse	CCACACAGTTGCTCCACAT
***Pfkfb3***	Forward	GGAGGTCGGCATGTTGAAGA
	Reverse	CTTTGGAAGGGCCTGAGAGG
***Muc2***	Forward	CGACTGTGAGCAGTGTGTCT
	Reverse	GGGTAGGGTCACCTCCATCT
***Tff3***	Forward	TGCAGATTACGTTGGCCTGT
	Reverse	TGCAGAGGTTTGAAGCACCA
***Cldn1***	Forward	CACTCCCAGACTCCACCACC
	Reverse	CGATCCATCCCAGAGAAGCC
***B2m***	Forward	CCCAGTGAGACTGATACATACG
	Reverse	CGATCCCAGTAGACGGTCTTG

### 2.16. 16S rRNA gene processing and analysis

DNA was extracted from colon luminal content using the PureLink™ Microbiome DNA Purification Kit from Thermo Fisher. The V3-V4 variable regions of the 16S rRNA gene were amplified and sequenced as 250-bp paired-end reads. The nf-core/ampliseq workflow version 2.11.0 was used to process the generated data.^31–33^ Briefly, the quality of the data was evaluated with FastQC (Andrews, 2010 - https://www.bioinformatics.babraham.ac.uk/projects/fastqc/.) and summarized with MultiQC.^34^ DADA2 was used to process the sequences^35^ eliminating PhiX contamination, discarding reads with more than 2 expected errors, correcting errors, merging read pairs, and removing PCR chimeras. Taxonomic classification was performed by DADA2, using the Silva 138.1 prokaryotic SSU database.^36^ The amplicon sequencing variants (ASVs) sequences, abundance, and taxonomic assignments were loaded into QIIME2^37^ and the ASVs with taxonomic strings containing mitochondria, chloroplast, or archaea were removed. Within QIIME2, the microbial community data was visualized in barplots and evaluated for sufficient sequencing depth with alpha rarefaction curves. Final visualizations were obtained as bar plots with the R libraries phyloseq,^38^ microViz^39^ and MicrobiotaProcess.^40^ After rarefaction, Shannon’s index was used to assess alpha diversity, and Bray-Curtis distances were used to assess beta diversity. LEfSe (Linear discriminant analysis Effect Size) analysis was performed using the run_lefse function from the microbiomeMarker R package. The analysis was conducted at the genus taxonomic level with the statistical significance thresholds set as follows: Kruskal-Wallis test cutoff (kw_cutoff) of 0.05 to detect features with significant differences among classes, Wilcoxon rank-sum test cutoff (Wilcoxon_cutoff) of 0.05 for pairwise comparisons, and an LDA score cutoff (lda_cutoff) of 2.0 to estimate the effect size of each differentially abundant taxon.

### 2.17. Proteomic analysis

Colon epithelial cells from wild-type and knockout mice (*n* = 10) were submitted to protein extraction using lysis buffer (100 mM Tris HCL, 150 mM NaCl, 1 mM EDTA, 1% Triton X) with freshly added protease inhibitors (cOmplete Mini Protease Inhibitor Cocktail, Roche). Tissue samples were incubated in lysis buffer for 30 min on ice before mechanical lysis by maceration, followed by ultrasonication (3 cycles of 20s, output 1.3) with a probe ultrasonic homogenizer (Cole-Parmer). Membranes and cellular debris were pelleted by centrifugation at 2000 x *g* for 10 min at 4°C and quantified using a microBCA kit (Thermo Fisher Scientific) with bovine serum albumin as the quantification curve. 20 micrograms of protein were washed, reduced, alkylated and digested using a modified filter-aided sample preparation (FASP) protocol.^41^

Digested peptides from each sample were desiccated by SpeedVac vacuum concentrator (Eppendorf) and resuspended in 0.1% formic acid (FA). The separation of tryptic peptides was performed on an ACQUITY M-Class System (Waters Corporation). One microgram of each digested sample was loaded onto a Symmetry C18 trapping column (5 μm, 180 μm × 20 mm; Waters Corp.) and subsequently separated by a 120-min reversed phase chromatography method at 300 nL/min (linear gradient, 3–55% ACN over 90 min) using a HSS T3 C18 analytical column (1.8 μm, 75 μm × 150 mm; Waters Corp.) maintained at 40°C. For the gradient elution, water: formic acid (99.9:0.1, v/v) was used as eluent A and acetonitrile: formic acid (99.9:0.1, v/v) as eluent B. The separated peptides were analyzed by a Synapt G2-S*i* mass spectrometer directly coupled online to the chromatographic system. The eluting peptides were ionized by nano-electrospray ionization in positive mode (nESI+) with a capillary voltage of 2.5 kV. Peptide and fragment ions were acquired using full-spectrum (m/z 50–2000), data-independent acquisition of ions with alternating low (4 eV) and high (ramping 25–60 eV) energies in the collision cell with ion mobility separation (HDMSe) with a scan time of 1.0 s. Ion mobility separation was performed with a wave velocity of 1000 m/s and transfer wave velocity of 175 m/s. A time-aligned inventory of accurate mass flight times in the TOF separation chamber was performed using a reference lock mass ([Glu1]-Fibrinopeptide B Standard, Waters Corp.). Each sample was run in three or four aligned instrumental replicates to improve precursor identification.

Continuum LC-MS data from three replicate experiments for each sample were processed for peak picking, retention time alignment, normalization, and protein identification and quantitation using Progenesis QI for Proteomics (Version 4.0.6x; Waters Corp.). The protein identification was performed using a database search algorithm against the reviewed *Mus musculus* database (UNIPROT Proteome ID UP000000589, retrieved June 2021, 17085 hits). Search parameters were set as: tolerance of 20 ppm for precursor ions and 10 ppm for product ions, maximum protein mass of 400 kDa, minimum 1 fragment ion matched per peptide, minimum 3 fragment ions matched per protein, minimum 1 unique peptide matched per protein, up to 2 missed cleavages, carbamidomethylation of cysteine as a fixed modification and oxidation of methionine as a variable modification, and 1% for false discovery rate (FDR), calculated using a reverse database library generated on-the-fly.

Label-free quantitative data were obtained using the relative abundance intensity integrated into Progenesis software, using all identified peptides for normalization. The expression analysis was performed considering technical replicates available for each experimental condition following the hypothesis that each group is an independent variable. Filtered tables were generated to include proteins found at least in two out of three technical replicates and to exclude proteins showing less than 20% change and those showing no statistical significance (ANOVA ≥0.05).

Downstream proteomics data analyses, including differential proteomic and enrichment analyses, were performed using OmicScope.^42^ For enrichment analysis, we searched differentially regulated proteins against Biological Process – Gene Ontology database. Figures were generated with the OmicScope web application (https://omicscope.ib.unicamp.br.) and Cytoscape environment.^43^

### 2.18. Statistical analysis

Except for proteomic, scRNA-seq and 16s rRNA data, all statistical analyses were performed using GraphPad Prism 8.0 software. The results are presented as mean and standard deviation. Comparisons between two groups were conducted using unpaired two-tailed t-tests. For multiple group comparisons, one-way or two-way ANOVA was applied, as appropriate. Details of the statistical tests used are provided in the figure legends. Statistical significance was set at *p* < 0.05.

## 3. Results

### 3.1. Inulin ingestion triggers distinct transcriptional changes in colonic enterocytes and proliferating cells

Our previous study highlighted inulin’s capacity to remodel the colonic epithelium through complex interactions between the microbiota and immune system.^11^ In the original study, we generated a single-cell RNA sequencing dataset to examine shifts in the proportions of intestinal epithelial subtypes and the expression of cell cycle – related genes. Here, to assess the impact of a high-fiber diet, we re-analyzed the data,^11^ comparing mice fed an inulin-supplemented diet versus a cellulose-fiber control diet, and evaluated transcriptional changes across epithelial cell subsets. For this analysis, we retained the original cell annotations and dimensionality reduction, as described in the original publication.^11^

Inulin intake significantly influenced gene expression across absorptive (AC; 1,379 genes), proliferative (PC; 761 genes), and secretory (SEC; 243 genes) intestinal cell populations (Figure S1A-C). Although some DEGs, particularly those that were downregulated (Figure S1B), were shared among the epithelial populations, the majority of DEGs were unique to each cell type, suggesting that the inulin diet exerts cell-type-specific effects on IECs. Enrichment analysis using the KEGG database revealed that inulin-induced genes were associated with the ‘protein processing in the endoplasmic reticulum’ pathway, which was common to both SECs and ACs (Figure S1C). In ACs, other pathways were found to be enriched, including ‘RNA transport’ and metabolic pathways, namely ‘alanine, aspartate, and glutamate metabolism,’ ‘propanoate metabolism,’ and the ‘citrate cycle’ (Figure S1C). Conversely, downregulated genes were linked to terms such as ‘thermogenesis’ and ‘oxidative phosphorylation’ in PCs and SECs, while the genes in ‘ribosome’ were consistently repressed across all cell populations (Figure S1C). These results indicate that the inulin diet drives distinct transcriptional profiles in different epithelial cell types, potentially affecting key biological processes in the gut.

**Figure 1.**
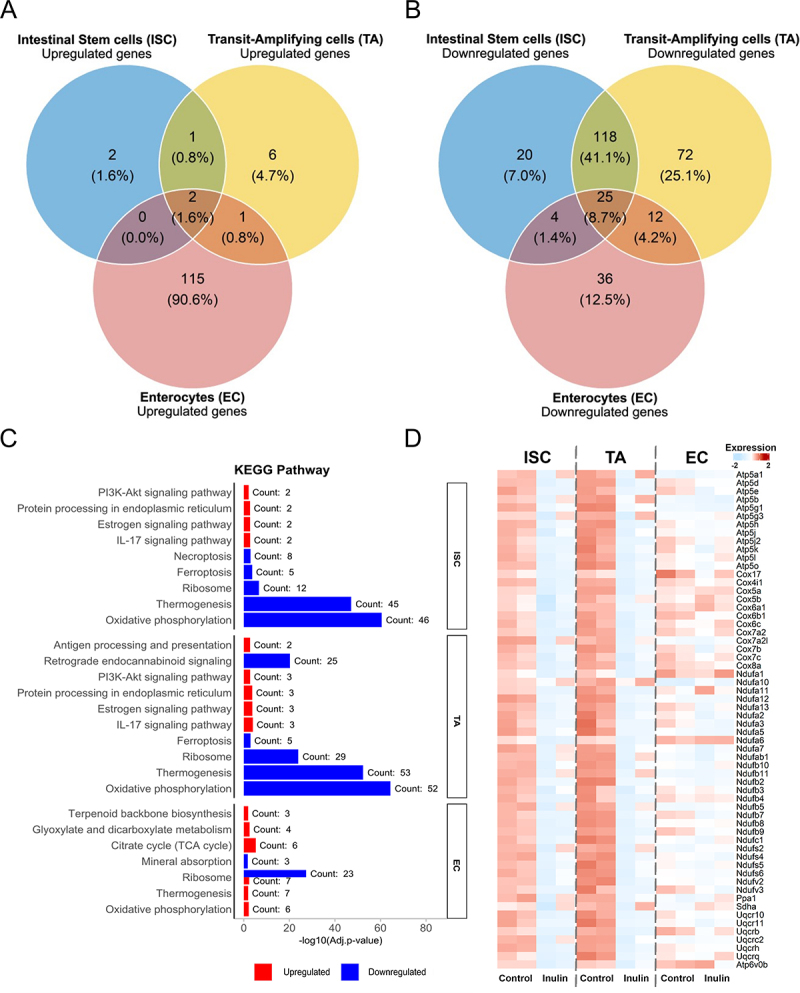
Inulin ingestion induces distinct transcriptional profiles in enterocytes and proliferating cells in the colon. (A) Venn diagram comparing the upregulated genes among intestinal stem cells (ISC), transit-amplifying cells (TA) and enterocytes (EC) obtained from the scRNA-seq analysis of intestinal epithelial cells (IECs) in inulin-treated animals. (B) Venn diagram comparing the downregulated genes among ISC, TA and EC obtained from the scRNA-seq analysis of IECs in inulin-treated animals. (C) KEGG pathway analysis by enrichR highlighting the top five terms for the up- (red) and downregulated (blue) genes among ISC, cycling TA and EC obtained from the scRNA-seq analysis of IECs in inulin-treated mice. (D) Average expression of selected oxidative phosphorylation (OXPHOS) genes in ISC, TA, and EC populations from control and inulin-fed mice. Genes were selected from significantly enriched pathways identified by gene ontology analysis. Color scale represents Z-score normalized expression values.

To better clarify these findings, we analyzed DEGs and functional terms within specific subsets of intestinal epithelial cells, uncovering contrasting effects of inulin-diet between enterocytes (ECs), which make up over 60% of the cells in our dataset, and the two main proliferative subtypes: cycling transit- amplifying cells (TAs) and ISCs. A comparison of the DEGs across these three IEC subsets revealed that most upregulated genes were specific to enterocytes (Figure 1A), whereas a significant portion of the genes downregulated by inulin were shared between TAs and ISCs (approximately 50% of the total DEGs), with a much smaller proportion ( < 15%) shared with enterocytes (Figure 1B). This data suggests that inulin has markedly different effects on gene expression in ECs compared to progenitor and proliferative cell populations. This difference becomes even more evident when looking at the KEGG terms related to the DEGs. Although inulin uniformly suppressed ‘ribosome’ genes across all cell subtypes, its effect on key metabolic pathways varies. In the EC compartment, an enrichment of tricarboxylic acid cycle (TCA) and oxidative phosphorylation pathways was observed in inulin-fed mice. In contrast, genes of oxidative phosphorylation were significantly downregulated in intestinal ISCs and TAs (Figure 1C,D). Inulin diet reduced the expression of genes encoding the alpha subunit of ATP synthase F1, subunits of the cytochrome c oxidase complex (Cox), and components of complex I of the mitochondrial oxidative phosphorylation chain in ISCs and TAs (Figure 1D). These changes were not observed in ECs (Figure 1D). Together, these findings suggest that inulin intake alters intestinal epithelial cell metabolism, potentially reducing oxidative metabolism in ISCs and transit-amplifying cells, while increasing it in enterocytes. This is consistent with the elevated butyrate production observed in mice fed with an inulin-rich diet^11,44,45^ and may reflect the zonation/distribution of these bacterial-derived metabolites along the crypt axis.^46^

### 3.2. Inulin diet amplifies intestinal hypoxia and stabilizes HIF-1 in gut epithelial cells

Inulin fermentation by the gut microbiota generates several bacterial metabolites, including SCFAs, which are essential for maintaining epithelial barrier integrity and metabolic homeostasis.^47,48^ Among them, butyrate has been shown to induce intestinal hypoxia by increasing mitochondrial-dependent oxygen consumption by IECs, which in turn stabilize HIF-1, a master cellular oxygen sensor that plays a key role in the maintenance of barrier function in the colon.^20^

**Figure 2.**
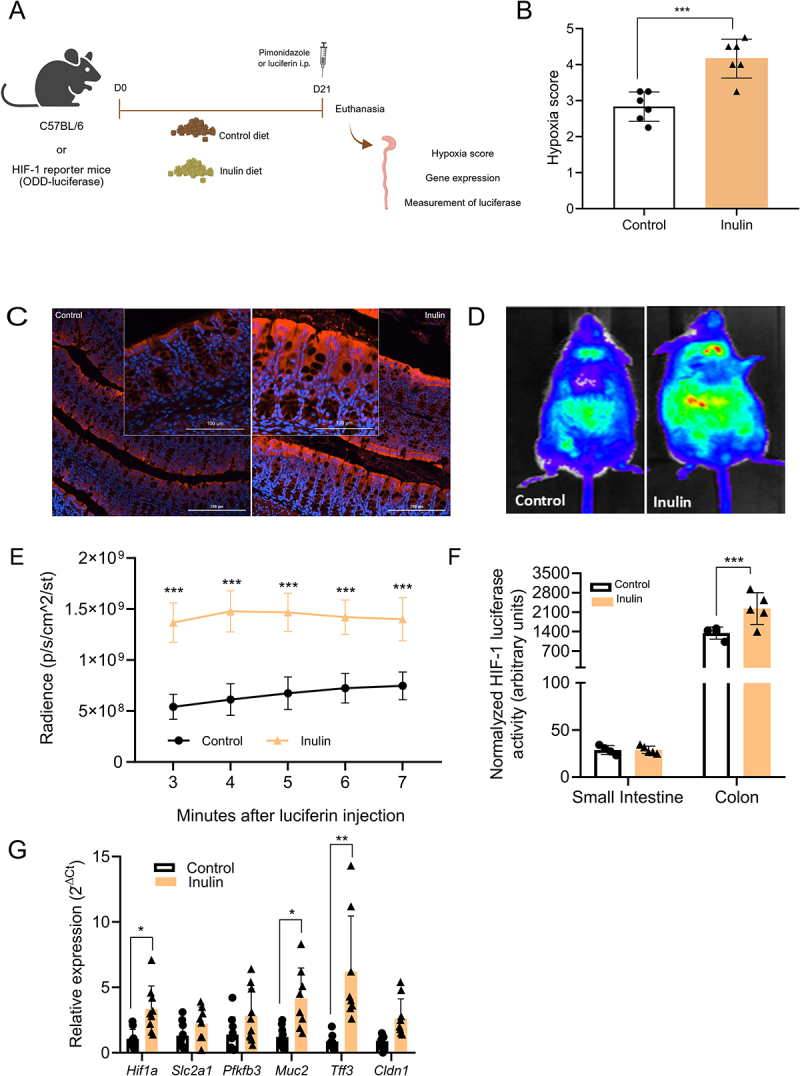
Inulin ingestion increases intestinal hypoxia and HIF-1a stabilization in intestinal epithelial cells. (A) Experimental design illustrating diets treatments and analyses performed in mice. (B) PMDZ staining was quantified by scoring sections of proximal colon from conventional mice fed with a control or a 10% inulin diet (*n* = 6). (C) Representative images of colonic sections stained with PMDZ from conventional mice treated with control and inulin diet for 3 weeks. Bars = 200 µm, 20x magnification. Zoom insets were taken from different areas or sections. Scale bars in the insets represent 100 µm (D) Representative images captured on the IVIS-SPECTRUM equipment 5 min after luciferin injection in ODD-mice fed with control or inulin diets (*n* = 3–5). (E) Luminescence quantification over time after luciferin injection in mice fed with control and inulin diet (*n* = 3–5). (F) Normalized HIF-1 luciferase activity in the small intestine and colon from mice treated with control or inulin diet (*n* = 3–5). (G) Relative quantification of *Hif1a* and its target genes expression on colonic epithelial cells by RT-qPCR (*n* = 8). Data were analyzed using Student’s *t*-test. In all graphs, each point represents an individual mouse. *p < 0.05, **p < 0.01, ***p < 0.001.

Given this, we next investigated whether inulin ingestion influences intestinal epithelial cells by modulating intestinal hypoxia and the activity of HIF-1. To do so, mice were fed for three weeks with the control or inulin-enriched diets (Figure 2A). Animals exhibited no differences in food intake or body weight (data not shown). Gut hypoxia was assessed using PMDZ staining. PMDZ is a 2-nitroimidazole that is specifically activated in hypoxic cells and tissues, forming stable complexes with thiol groups on proteins, peptides, and amino acids. The intake of the inulin diet increased colonic hypoxia, with greater PMDZ intensity and deeper distribution along the crypt axis compared to controls (Figure 2B,C).

Next, we assessed HIF-1 stabilization using the ODD-luciferase transgenic mice, which express luciferase under the control of the oxygen-dependent degradation domain of HIF-1α.^49^ Following luciferin administration, inulin-fed mice exhibited stronger chemiluminescence in the abdominal area compared to controls, indicating increased HIF-1α stabilization (Figure 2D–E). In inulin-fed mice, we also observed a signal originating from a region located above the lungs, the nature of which will require further investigation (Figure 2D). After euthanasia, luminescence was higher in colonic epithelial cells than in those from the small intestine, consistent with the gastrointestinal oxygen gradient^14^ (Figure 2F). Additionally, colonic IECs from inulin-fed mice showed greater HIF-1α stabilization than controls (Figure 2G). This effect was accompanied by elevated expression of *Hif1a* and some HIF-1 target genes, including mucin-2 (*Muc2*) and trefoil factor-3 (*Tff3*) in colonic epithelial cells (Figure 2G). Together, these data indicate that inulin ingestion intensifies colonic hypoxia, leading to increased HIF-1α expression and activity in epithelial cells. The partial upregulation of HIF-1 target genes suggests a limited response, potentially modulated by secondary regulatory mechanisms.

### 3.3. The effects of inulin on colonic hypoxia and HIF-1 stabilization depend on both gut microbiota and epithelial PPARγ

**Figure 3.**
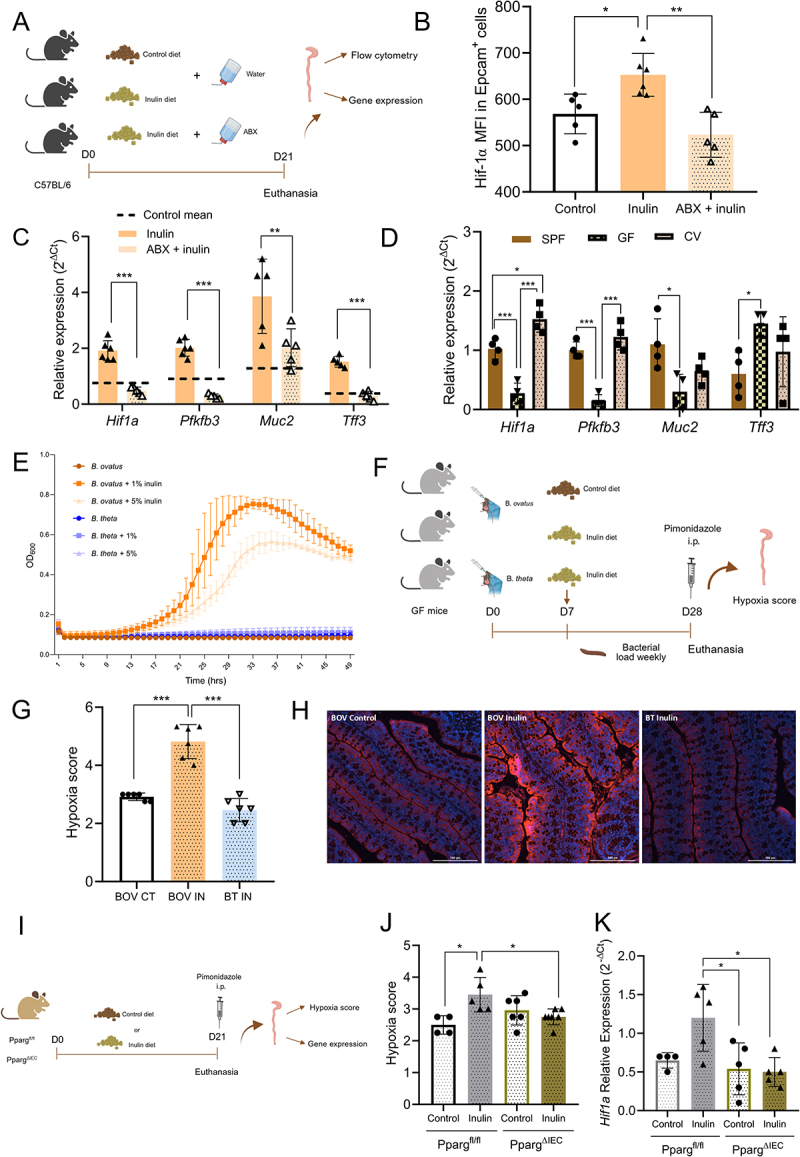
HIF-1α stabilization in response to inulin diet is dependent on colon microbiota and epithelial Pparg. (A) Experimental design illustrating diet and ABX treatments in C57BL/6 mice. The ABX mix consisted of 1 g/L ampicillin, 1 g/L neomycin, 1 g/L metronidazole, and 0.5 g/L vancomycin. (B) Median fluorescence intensity (MFI) for HIF-1α^+^ labeling in CD45^−^/EpCAM^+^ cells, as determined by flow cytometry, in mice fed control or inulin diet and treated with a mix of antibiotics (ABX + inulin) (*n* = 5). (C) Relative expression of *Hif1a* and HIF-1α target genes in mice fed with an inulin diet and treated with an antibiotic mix (*n* = 5). (D) Relative expression of *Hif1a* and HIF-1α target genes in specific pathogen-free (SPF), germ-free (GF) and conventionalized (CV) mice fed with a normal chow diet (*n* = 4). (E) *In vitro* growth curves of BOV and BT in media containing different amounts of inulin (*n* = 3). (F) Experimental design illustrating gnotobiotic mice colonized with *B. ovatus* (BOV) or *B. thetaiotaomicron* (BT), fed a control or inulin diet, followed by pimonidazole injection. (G) Hypoxia score in monocolonized mice fed control or inulin diet (*n* = 6). (H) Representative images of colonic sections stained with PMDZ from monocolonized mice treated with control and inulin diet for 3 weeks. Bars = 200 µm, 20x magnification. (I) Experimental design illustrating diets treatments and analyses performed in Pparg^fl/fl^ and Pparg^∆IEC^ mice. (J) Hypoxia score in Pparg^fl/fl^ and Pparg^ΔIEC^ fed with control or inulin diet (*n* = 4–7). (K) Relative mRNA expression of *Hif1a* in colonic epithelial cells from Pparg^fl/fl^ and Pparg^ΔIEC^, as determined by RT-qPCR. (*n* = 4–7). Data were analyzed using one-way ANOVA followed by Tukey’s multiple comparisons test (B and D), Student’s *t*-test (C) or two-way ANOVA followed by Sidak’s multiple comparisons test (J and K). In all graphs, each point represents an individual mouse. *p < 0.05, **p < 0.01, ***p < 0.001.

Next, we investigated the role of the gut microbiota in mediating the effects of inulin-diet on the colonic epithelium, as fermentation of soluble fibers relies on the intestinal bacterial community. For that, mice were fed an inulin-rich diet and treated with a cocktail of broad-spectrum antibiotics (ABX) to deplete the gut microbiota^25^ (Figure 3A). The depletion of microbiota was confirmed by the reduced bacterial load in feces of mice treated with the ABX cocktail (Figure S2A). ABX treatment reduced HIF-1α levels in IECs from inulin-treated mice (Figure 3B and S2B), as well as the expression of *Hif1a* and some of its target genes (Figure 3C). To further examine the microbiota’s impact on HIF-1, we conducted experiments with germ-free (GF), specific pathogen-free (SPF), and conventionalized (CV) mice. GF mice exhibited lower *Hif1a* expression and reduced levels of its target genes in colonic epithelial cells (Figure 3D). Conventionalization via cecal content gavage and concurrent co-housing with SPF mice restored the expression of *Hif1a* and some target genes (Figure 3D). These findings demonstrate that microbiota is necessary for regulating intestinal hypoxia and increasing *Hif1a* expression and its activity in response to inulin intake.

To better understand the role of inulin fermentation in our system, we conducted experiments using monocolonized mice. For this, we selected two individual bacterial species, *Bacteroides ovatus* (BOV) and *Bacteroides thetaiotaomicron* (BT). These species exhibited distinct growth capacities in media in which inulin was the sole carbon source (Figure 3E). This is consistent with previous reports showing that BOV can metabolize inulin, whereas BT cannot^50^. Monocolonized mice were kept on either a control or inulin diet for 3 weeks and the hypoxia levels of the proximal colon were measured using the PMDZ technique (Figure 3F). Both mice groups exhibited similar levels of bacterial colonization, indicating that BOV and BT have comparable capacities to colonize the gnotobiotic mouse colon (Fig S2C). Nonetheless, increased colonic hypoxia was only observed in mice monocolonized with BOV and fed with inulin (Figure 3G-H), indicating that inulin metabolism is mandatory for inducing intestinal hypoxia and, therefore, HIF-1 activity.

Previous studies by our group and others have shown that inulin ingestion increases butyrate production,^11,51,52^ a key metabolite that regulates various epithelial barrier functions.^11,20,53^ Butyrate promotes β-oxidation in epithelial cells via activation of peroxisome proliferator-activated receptor gamma (PPARγ), enhancing oxygen consumption and maintaining intestinal hypoxia.^16^ To investigate whether PPARγ plays a significant role in the inulin-induced epithelial hypoxia, we studied littermate mice with epithelial-specific deletion of *Pparg* (Pparg^fl/fl^ x VilCre^+^, Pparg^ΔIEC^) and wild-type controls (Pparg^fl/fl^ x VilCre^−^, Pparg^fl/fl^) (Figure 3I). After three weeks on either a control or inulin diet, we assessed intestinal hypoxia. As expected, Pparg^fl/fl^ mice on the inulin diet showed a significant increase in intestinal hypoxia evaluated by PMDZ staining and *Hif1a* expression in IECs (Figure 3J–K). However, these effects were absent in Pparg^ΔIEC^ mice, indicating that the PPARγ is directly involved in the development of hypoxia and subsequent increase in HIF-1α following inulin intake. Together, these findings suggest that inulin fermentation by the microbiota produces metabolites that activate PPARγ, driving mitochondrial β-oxidation and oxygen consumption, leading to increased intestinal hypoxia.

### 3.4. HIF-1 regulates colon epithelial cell proliferation in response to the inulin diet

**Figure 4.**
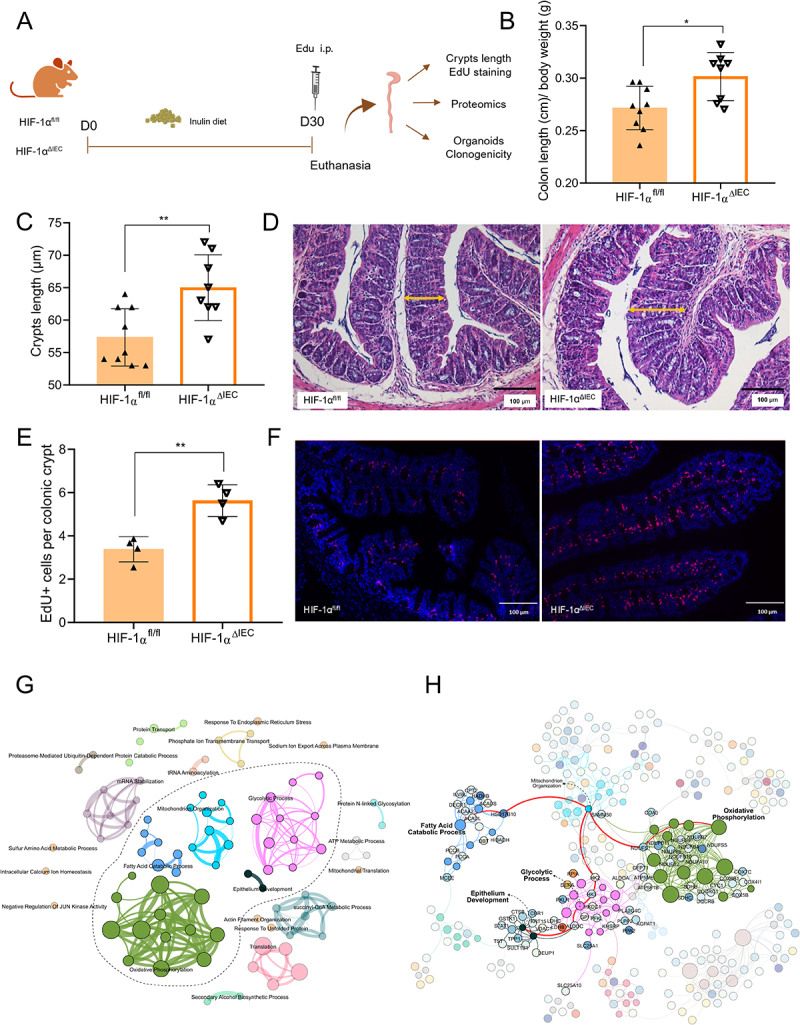
Epithelial deletion of HIF-1α increases intestinal epithelial proliferation and modulates metabolism pathways in response to inulin diet. (A) Experimental design illustrating diets treatments and analyses performed in HIF-1α^fl/fl^ and HIF-1α^∆IEC^ mice. (B) Colon length normalized by body weight in HIF-1α^fl/fl^ and HIF-1α^∆IEC^ mice fed with inulin diet (*n* = 8–9). (C) Crypts length normalized by body weight in HIF-1α^fl/fl^ and HIF-1α^∆IEC^ mice fed with inulin diet (*n* = 8–9). (D) Representative images of colonic sections stained with H&E from mice fed with inulin diet for 3 weeks. Bars = 100 µm, 20x magnification (*n* = 8–9). (E) Quantification of EdU-positive cells per crypt (*n* = 4). (F) Visualization of EdU-positive cells in colonic crypts by fluorescence microscopy following EdU Click-iT reaction . Bars = 100 µm, 20 ×magnification. (G) Biological process enrichment map from differentially regulated proteins (HIF-1α^ΔIEC^ vs HIF-1α^fl/fl^) in mice fed with inulin diet, highlighting the modules associated with cell metabolism (*n* = 5). (H) Process-protein network showing proteins differentially regulated (blue-red gradient for down- and upregulation, respectively) across metabolic modules. The red edges highlight key proteins and processes linking metabolic effects with epithelial development. Data were analyzed using Student’s *t*-test. In all graphs, each point represents an individual mouse. *p < 0.05, **p < 0.01.

HIF-1 is highly expressed in intestinal epithelial cells, where it regulates genes related to antimicrobial production, barrier function, mucin secretion, and energy metabolism.^14^ Given the stabilization of HIF-1α in response to the inulin diet, we next sought to evaluate the biological significance of this effect on the intestinal epithelium. To do so, we utilized mice with a targeted deletion of *Hif1a* in intestinal epithelial cells (HIF-1α^fl/fl^ x VilCre^+^, HIF-1α^ΔIEC^) alongside floxed littermate controls without Cre recombinase (HIF-1α^fl/fl^ x VilCre^−^, HIF-1α^fl/fl^) (Figure 4A). HIF-1α deletion in HIF-1α^ΔIEC^ mice was confirmed by quantitative PCR analysis of colon tissues from animals on the inulin diet (Figure S3A) and by assessing HIF-1α levels via flow cytometry (Figure S3B and S3C). No differences were observed in body weight, small intestine or cecum lengths (Figure S3D-F) in HIF-1α^ΔIEC^ mice when compared to littermate controls. Nonetheless, when maintained on inulin diet, HIF-1α^ΔIEC^ mice displayed increased colon length and deeper intestinal crypts compared to controls (Figure 4B–D). Moreover, there was a marked increase in proliferative cells, as shown by EdU (S phase) and Ki67 (G1, S, G2, and M phases) staining (Figure 4E,F, and S3G-I). Additionally, we demonstrated that the increment on intestinal proliferative cells and crypt size in HIF-1α^ΔIEC^ mice, compared to HIF-1α^fl/fl^ mice, was dependent of the inulin-diet, as HIF-1α^ΔIEC^ mice on a control diet did not present this response (Figure S3J). We also analyzed the gut microbiota composition of these mice and found that, although there were no significant changes in overall community structure, as assessed by alpha and beta diversity (Figure S4A – C), LEfSe revealed specific taxonomic shifts, including an increased abundance of the *Muribaculaceae* family in HIF-1α^ΔIEC^ mice (Figure S4D). Together, these data indicate the interplay between diet and the gene regulatory machinery involving HIF-1 in regulating the colonic epithelium.

To investigate the mechanism behind the increased proliferation in HIF-1α^ΔIEC^ mice, we performed an unbiased proteomic analysis of IECs from HIF-1α^ΔIEC^ and their littermates on the inulin diet (Figure 4G–H, and S5). This analysis revealed differential regulation of approximately one-fourth of the identified proteins (435 out of 1601), with 361 downregulated and 74 upregulated in HIF-1α^ΔIEC^ compared to HIF-1α^fl/fl^ mice (Figure S5). Biological process enrichment map from differentially regulated proteins (HIF-1α^ΔIEC^ vs HIF-1α^fl/fl^) in mice fed with inulin highlighted important modulation of modules associated with cell metabolism including mitochondrial organization, fatty acids catabolic processes and glycolytic processes in addition to modulation of epithelium development (Figure 4G). Furthermore, process-protein network showed proteins differentially regulated across metabolic modules. Some proteins involved in glycolytic process such as aldolases (Aldoa and Aldoc) and hexokinases (Hk2–3 and Hkdc1), oxidative phosphorylation such as NADH dehydrogenase [ubiquinone] iron-sulfur family (Ndufs subunits 4, 7, 9, 10 and 11) and cytochrome c oxidase family (COX) and fatty acid catabolic process such as carnitine palmitoyltransferase 2 (Cpt2) and short-chain specific acyl-CoA dehydrogenase (Acads) were seen downregulated in HIF-1α^∆IEC^ epithelium (Figure 4H). On the other hand, most of the upregulated proteins in HIF-1α^ΔIEC^ epithelium were associated with mitochondrial carrier protein family (Timm23, Timm50, Mtch1 and Gfm1), glycolytic process and cellular response to stress (Rpia and Dera, respectively), besides cell cycle control proteins (Cdc42). Interestingly, most of these differentially regulated proteins link metabolic effects with epithelial development, consistent with an important role of HIF-1 in regulating metabolic pathways, which in turn modulate the proliferation and differentiation of the intestinal epithelium.

### 3.5. HIF-1 regulates stem cell activity in response to inulin diet

**Figure 5.**
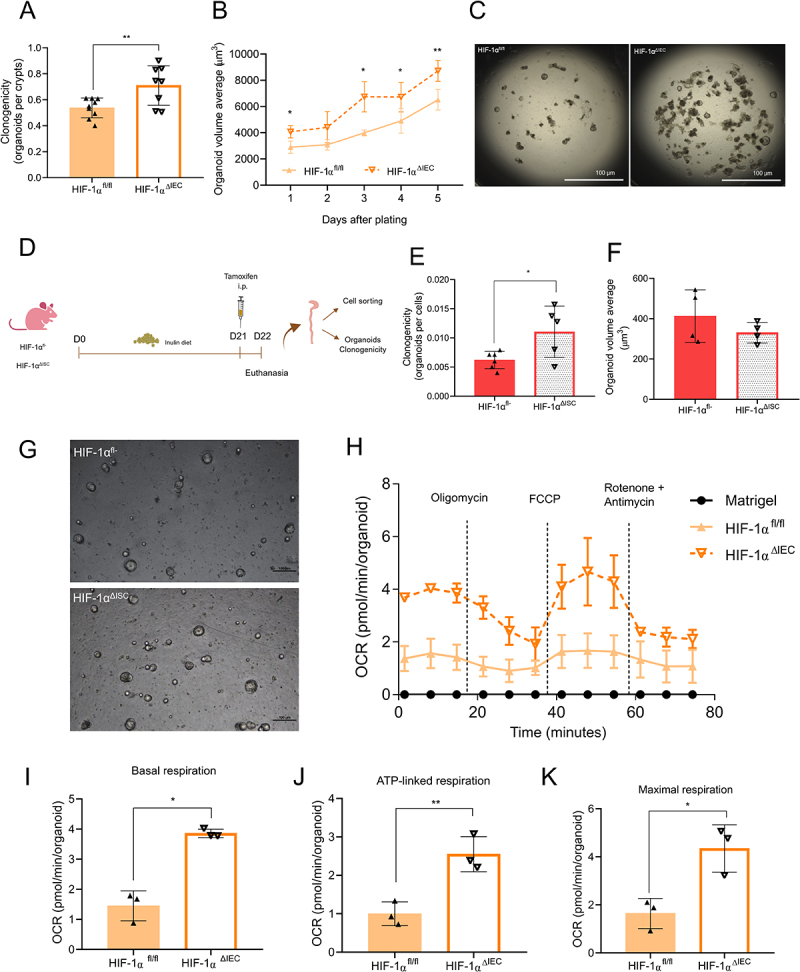
HIF-1α plays a critical role in intestinal stem cell (ISC) function by modulating oxidative metabolism. (A) Quantification of the clonogenicity capacity of colon crypts in mice with and without epithelial deletion of HIF-1α (*n* = 9). (B) Growth kinetics of organoids (*n* = 9). (C) Representative images of organoids from mice with HIF-1α epithelial deletion. (D) Experimental design for sorting cell experiments with HIF-1α^ΔISC^ mice (*n* = 5–6). (E) Clonogenicity capacity of sorted-ISCs from HIF-1α^ΔISC^ mice and their controls (*n* = 5–6). (F) Organoid volume average from HIF-1α^ΔISC^ mice and their controls (*n* = 5–6). (G) Representative images of organoids from HIF-1α^ΔISC^ mice and their controls (*n* = 5–6). (H) Oxygen consumption rates (OCRs) of organoids, as measured by CellMito stress test and a Seahorse XF extracellular flux analyzer (*n* = 3). (I) Basal respiration of colonic organoids (*n* = 3). (J) ATP-linked respiration of colonic organoids (*n* = 3). (K) Maximal respiration of colonic organoids (*n* = 3). Data were analyzed using Student’s *t*-test. In all graphs, each point represents an individual mouse. *p < 0.05, **p < 0.01.

Intestinal epithelial barrier homeostasis relies on the balance between self-renewal and differentiation of ISCs, processes regulated by both intrinsic and extrinsic signals including diet and microbiota.^54^ Our previous study showed that inulin ingestion increases the proliferation and activity of colonic stem cells compared to a control diet.^11^ Based on these findings, we tested if HIF-1 is involved in these effects of inulin specifically on ISCs. HIF-1α^ΔIEC^ mice and their floxed controls were fed an inulin diet, followed by colonic crypts isolation for clonogenicity assay, according to the protocol by Beyaz et al. (2016).^55^ We found that the conversion of crypts into organoids was significantly higher in knockout animals compared to their littermates (Figure 5A and C), which also exhibited larger volume and growth (Figure 5B), thus indicating stronger ISCs activity in the absence of HIF.

To further validate the role of HIF-1α, we performed a similar analysis on von Hippel-Lindau tumor suppressor (VHL)-deficient mice (VHL^ΔIEC^) (Figure S6A). The VHL protein regulates the stability of HIF-(1/2)α, and its deletion leads to constitutive HIF-1α stabilization in IECs by inhibiting its proteasomal degradation.^56^ VHL^ΔIEC^ mice presented no significant differences in body weight, colon size, or colon crypt length (Figure S6B-D). However, consistent with the results from HIF-1α-deficient mice, VHL^ΔIEC^ mice without inulin treatment exhibited reduced epithelial cell proliferation, as observed by a significant lower number of EdU-positive cells (Figure S6E). Similarly, VHL^ΔIEC^ crypts showed reduced clonogenicity capacity but with no changes in organoid volume (Figure S6F-G), indicating that constitutive expression of HIF-1 impairs ISCs activity.

We next performed experiments using an ISC-specific HIF-1α-deficient mouse model (Lgr5-Cre; Ai14; HIF-1α^fl/fl^, termed HIF-1α^ΔISC^) to confirm whether the effect of HIF-1α on organoid formation was intrinsic to ISCs. Both HIF-1α^ΔISC^ mice and their controls (Lgr5-Cre; Ai14; HIF-1α^fl/-^, termed HIF-1α^fl/-^) were maintained on an inulin diet during the entire protocol and received tamoxifen (i.p.) 24 hours before the experiment to induce tomato fluorescence (Figure 5D). We then FACS-sorted viable cells with high tdTomato signal and plated them for organoid formation (Figure 5D). We found that organoids formation was more efficient in HIF-1α-deficient ISCs compared to controls expressing normal levels of HIF-1α (Figure 5E–G) confirming that HIF-1α regulates ISCs functions in response to inulin diet.

### 3.6. HIF-1α modulates intestinal epithelial proliferation through metabolic reprogramming

Since our proteomics data indicated that HIF-1α deletion leads to changes in the metabolic pattern of the intestinal epithelium (Figure 4G–H), we sought to determine the impact of its absence on mitochondrial function and, consequently, on the oxidative metabolism in intestinal organoids. The mitochondrial respiratory capacity of organoids from HIF-1α^ΔIEC^ mice and their controls was evaluated using a Cell Mito Stress Test on a Seahorse XF extracellular flux analyzer. We found oxygen consumption rates (OCRs), ATP-linked, and maximal mitochondrial respiration were significantly elevated in HIF-1α^ΔIEC^ organoids (Figure 5H–K). These changes were not accompanied by alterations in mitochondrial mass, which was examined by MitoTracker green (Figure S7A-D), indicating that the effect of HIF-1 deletion in the IECs is not dependent on the mitochondrial mass, but on mitochondrial activity.

**Figure 6.**
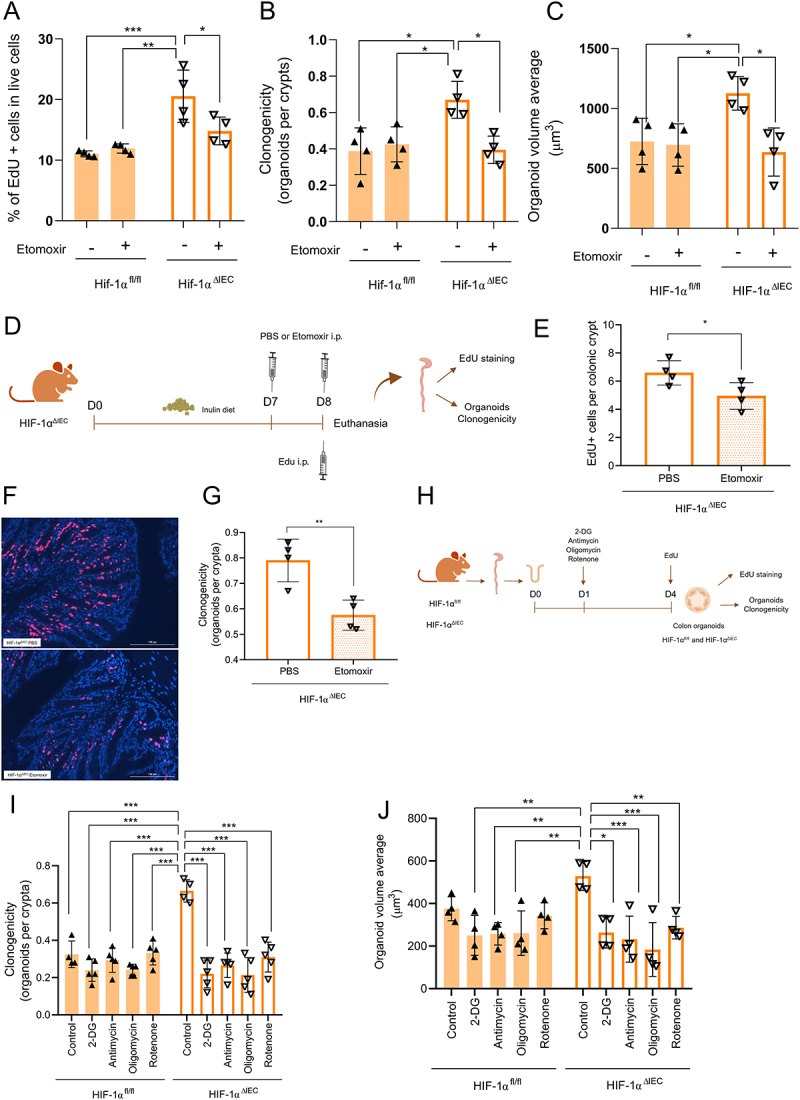
HIF-1 controls colon cell proliferation by regulating oxidative phosphorylation and mitochondrial function in ISCs. (A) Percentage of live EdU-positive cells in colon organoids after treatment with the FAO inhibitor, etomoxir (*n* = 4). (B) Clonogenicity capacity of colon crypts treated with etomoxir (*n* = 4). (C) Measurement of organoids volume after 4 days of etomoxir treatment (*n* = 4). (D) Experimental design of etomoxir *in vivo* treatment. (E) EdU-positive cells in colon sections from mice treated with etomoxir (20 mg/kg i.p.) (*n* = 4). (F) Representative images of EdU-positive cells in the colon of mice treated with PBS or etomoxir (*n* = 4). Bars = 100 µm, 20 ×magnification. (G) Clonogenicity capacity of colon crypts from mice treated with etomoxir (*n* = 4). (H) Experimental design of organoids treatment with different inhibitors of glycolysis and mitochondrial complexes. (I) Clonogenicity capacity of colon crypts treated with glycolysis inhibitor, 2-deoxyglucose (2-DG), and inhibitors of mitochondrial complexes (*n* = 3–5). (J) Organoid volume average from organoids treated with oligomycin, antimycin, rotenone or 2-DG (*n* = 3–5). Data were analyzed using two-way ANOVA followed by Sidak’s multiple comparisons test (A, B, C, I and J) or Student’s *t*-test (E and G). In all graphs, each point represents an individual mouse. *p < 0.05, **p < 0.01, ***p < 0.001.

Fatty acid oxidation (FAO) is an important source of intermediates for the TCA cycle and provides electron carriers (NADH and FADH_2_) that fuel the mitochondrial electron transport chain, leading to ATP production. This process has been linked with ISC stemness.^57^ To investigate if FAO is connected to the increased proliferation in HIF-1α^ΔIEC^ mice, we treated organoids from HIF-1α^ΔIEC^ and HIF-1α^fl/fl^ mice with low doses of etomoxir, an FAO inhibitor that targets carnitine palmitoyl transferase I (CPT-1).^58^ Once again, HIF-1α^ΔIEC^ derived organoids presented a higher number of proliferating cells, as assessed by EdU staining (Figure 6A), and HIF-1α^ΔIEC^ colonic crypts showed higher clonogenicity, forming larger organoids (Figure 6B,C) when compared to the control condition. These effects were abrogated in HIF-1α^ΔIEC^-derived organoids treated with etomoxir, while the drug had no effect in HIF-1α^fl/fl^ organoids. Furthermore, *in vivo* treatment with etomoxir also reduced colonic epithelial proliferation in HIF-1α^ΔIEC^ mice fed with inulin, as observed by reduced number of EdU-positive cells (Figure 6D–F), and a diminished ability of the crypts to generate organoids (Figure 6G). These data suggest that FAO contributes to the increased ISC proliferation and activity in the absence of HIF-1. Additionally, we examined how HIF-1α^ΔIEC^-derived organoids responded to mitochondrial inhibitors and the glycolysis inhibitor 2-deoxyglucose (2-DG) (Figure 6H). Treatment with 2-DG, antimycin (complex III inhibitor), oligomycin (complex V inhibitor), or rotenone (complex I inhibitor), at the concentrations used in our study, had no effect on the growth of HIF-1α^fl/fl^ organoids. However, these treatments impaired the phenotype observed in HIF-1α^ΔIEC^-derived organoids, which no longer showed any differences when compared to the control condition (Figure 6I,J and S7E). These findings highlight the crucial role of HIF-1 in regulating ISC function through metabolic reprogramming in the colon.

## 4. Discussion

We describe a previously unrecognized function of HIF-1α in regulating host-microbiota interactions, expanding its known functions in the intestinal epithelium, which include maintaining barrier integrity, inducing antimicrobial peptides, and modulating immune responses.^20^ We have previously shown that the ingestion of an inulin-enriched diet promotes epithelial proliferation via modulation of ISCs activity in a microbiota-dependent manner.^11^ Here, we now demonstrate that this proliferative phenotype is significantly amplified in mice with epithelial-specific deletion of HIF-1α. These mice exhibit elevated ISCs activity and markedly increased epithelial proliferation compared to their wild-type counterparts. Therefore, our findings suggest that, under homeostatic conditions, HIF-1α acts as a regulatory brake, fine-tuning the impact of the dietary fiber inulin on intestinal epithelial dynamics.

We link the role of HIF-1α in the colon epithelium to its impact on regulation of stem and proliferative cell activity. HIF-1 plays a pivotal role in regulating ISCs and modulating their responses under both physiological and pathological conditions by directly interacting with key signaling pathways essential for ISC function, including Wnt/β-catenin, Notch, and JAK/STAT pathways.^59–61^ Additionally, HIF-1 can indirectly influence ISC activity by acting on the stem cell niche within the intestinal crypts, promoting the release of growth factors and cytokines that stimulate proliferation and inhibit apoptosis.^62,63^ Some of the roles of HIF-1α in stem cells may be shared in other somatic stem cell compartments. A study of mouse muscle regeneration indicated that HIF1-signaling regulates stem cell proliferation and showed that disrupting the HIF signaling led to enhanced proliferation with potential depletion of the stem cell pool and reduced regenerative potential of the muscle and this has been also linked to the role of HIF in regulating Notch.^64,65^ Future research needs to illuminate shared and tissue-specific functions of HIF in regulating stem cell biology.

The regulatory role of HIF-1α in homeostasis contrasts with its established function in inflammation and tumorigenesis, where it typically enhances cell proliferation and survival.^66–68^ In such pathological settings, increased HIF-1α activity is often attributed to reduced oxygen availability in the tumor microenvironment^69^ and/or a metabolic shift known as the Warburg effect, in which cancer cells preferentially use glycolysis over oxidative phosphorylation even in the presence of oxygen.^70^ However, some studies have shown that the absence of HIF-1α can paradoxically stimulate growth and proliferation in colon cells.^71^ The conditional deletion of HIF-1α in IECs can lead to accelerated tumor growth in certain models of colorectal cancer, suggesting that HIF-1α can also play a role in inhibiting tumor progression.^72^ Therefore, together with the studies described above, our findings highlight that the effects of HIF-1 on the regulation of cell proliferation are context-dependent and influenced by factors such as the types of cells involved, the presence of inflammation or dysbiosis, and the metabolic state of the tissue.

We show that an inulin-rich diet promotes microbial fermentation, leading to increased intestinal hypoxia and activation of HIF-1 signaling in epithelial cells. This, in turn, regulates epithelial metabolism and influences ISC proliferation and maintenance. Prior studies have shown that the SCFA butyrate has a pivotal role in modulating intestinal oxygen levels.^20^ As a primary energy source for IECs, butyrate enhances mitochondrial oxidative phosphorylation, increases oxygen consumption, and contributes to a hypoxic microenvironment.^16^ This hypoxia is crucial for preventing dysbiosis and sustaining epithelial integrity.^14,16,21,73,74^ In our previous work, we demonstrated that inulin consumption increases the levels of acetate, propionate, and butyrate compared to a cellulose-based control diet.^11^ In this follow-up study, we indirectly link this SCFA enrichment to elevated intestinal hypoxia and enhanced HIF-1 signaling in IECs. While future metagenomic and metabolomic analysis are needed to identify the precise microbial metabolites involved, our data provide evidence that inulin, via microbial fermentation, differentially affects the metabolic activity of IEC subsets. Specifically, it suppresses oxidative phosphorylation in the proliferative compartments of the crypts, including on ISCs and TAs, while promoting it in differentiated enterocytes. Together, these findings support the concept of spatial gradients in both oxygen and microbial metabolites along the crypt axis, which drive distinct metabolic responses in the functionally and spatially diverse populations of IECs that make up the intestinal epithelium.^46,75,76^ Future studies should dissect the metabolic landscape across distinct epithelial cell populations, including mature, progenitor/proliferative and stem cells, which, as suggested by our transcriptomic data, exhibit differential metabolic responses to inulin. Single-cell metabolomic approaches will be particularly valuable in resolving the cell-type – specific changes.

Our proteomic analyses of epithelial cells with and without HIF-1 on inulin diet revealed significant alterations in structural proteins associated with the mitochondrial membrane, findings that were corroborated through functional assays using selective inhibitors of mitochondrial respiratory complexes. These results suggest that HIF-1 signaling is a key mediator linking energy metabolism to epithelial cell turnover. Furthermore, these findings highlight the pivotal role of mitochondria as hub that integrates metabolic signals and relays these into regulation of cellular proliferation. The important roles of mitochondria in generating energy, calcium storage and regulating cell death are well established. Less in known about the roles of mitochondria in regulating stem cell renewal and differentiation.^77,78^ In *Drosophila*, mitochondrial electron chain transport activity promotes ISC proliferation and epithelial homeostasis through the transcriptional regulator FOXO^79^. Other studies, also in *Drosophila*, implicated mitochondrial fusion dynamics in the regulation of ISC biology^80^. Recent studies have shown that mitochondria play a key role in regulating intestinal stem cell (ISC) maintenance and differentiation in the mouse intestine.^30,81^ How HIF-1 regulates mitochondrial activity and affects ISCs functions will require further analysis.

What might be the biological role, if any, of increased proliferation in the intestinal epithelium and deepening of the crypt? The crypt has been suggested to be an invagination that protects the intestinal stem cells from toxic activities derived from microbial metabolites, including butyrate.^46^ Therefore, it is tempting to speculate that the role of HIF-1 in regulating proliferative activity in the intestinal epithelium and crypt depth is linked to this protective role. One possible scenario might be that reactive oxygen species (ROS) play an important role in our observations. ROS are generated by oxidative phosphorylation of SCFAs and might trigger proliferation, e.g., via signaling pathways, possibly promoting protection of the stem cells from their detrimental activity, similar to what has been described in the *Drosophila* system.^82,83^ Indeed, NOX1-dependent redox signaling has been shown to promote colonic cell proliferation.^84^

Given this multifaceted regulation, it remains challenging to delineate the precise mechanisms through which HIF-1 modulates ISC behavior and, consequently, epithelial architecture in response to dynamic luminal cues such as dietary composition, energy availability, and microbiota-derived signals. Several potential limitations of our study should be considered. First, we have not identified the specific inulin diet-dependent microbial metabolites that influence proliferation in the colonic epithelium. While butyrate has been linked to intestinal hypoxia, other metabolites, such as propionate, may also contribute. Another aspect that needs further investigation is the spatial gradient of oxygen and metabolites along the crypt axis, which we speculate may induce differential effects across epithelial compartments. An additional limitation is that, although we demonstrate that metabolic reprogramming is involved in the phenotype observed following HIF-1α deletion, we did not elucidate the underlying mechanisms. In this context, factors such as mitochondrial activity and the potential role of reactive oxygen species (ROS) generation should be further explored. Nonetheless, despite the complexity of this regulatory landscape, our findings underscore the essential role of HIF-1 in maintaining ISC function and, by extension, intestinal epithelial homeostasis in response to inulin intake, serving as a molecular break preventing cell over proliferation. Future work should evaluate these findings in the context of colorectal cancer, e.g., using appropriate mouse models.

## 5. Conclusion

In conclusion, our data highlights the impact of inulin on intestinal epithelial hypoxia and intestinal stem cell metabolism. It also demonstrates a significant novel role of HIF-1 in limiting intestinal epithelial proliferation in response to microbiota-derived metabolites produced by high-fiber diets, thereby contributing to the maintenance of colonic epithelial homeostasis.

## Supplementary Material

Supplemental Material

## Data Availability

- Single-cell RNA sequencing data is deposited at NCBI BioProject: PRJNA856646 - 16S sequencing data is deposited at NCBI BioProject: PRJNA1188097. - The mass spectrometry proteomics data is deposited to the ProteomeXchange Consortium (http://proteomecentral.proteomexchange.org.) via the PRIDE partner repository^85^ with the dataset identifier PXD059757. Project DOI: 10.6019/PXD059757
