# Supplementary material for: HIF-1 attenuates high-fiber diet-mediated proliferation and stemness of colonic epithelium
Source: Gut Microbes. 2025 Aug 19;17(1):2543123. doi: 10.1080/19490976.2025.2543123 (PMC12369635; doi:10.1080/19490976.2025.2543123)
Supplement: Supplemental Material [file KGMI_A_2543123_SM5400.zip › kgmi-s-2025-0298-20250731041635/graphic/Castro_et_al_2025_Gut_microbes clean (1) Supp.docx]

**Figure S1**

**Fig. S1| Inulin affects different intestinal cell populations in mice colon.** Venn diagram comparing the up- (A) and downregulated (B) genes among the proliferative (PC), secretory (SEC), and absorptive (AC) intestinal populations obtained from the scRNA-seq analysis of intestinal epithelial cells (IECs) in mice fed inulin diet. (C) KEGG pathway analysis by enrichR highlighting the top five terms for the up- (red) and downregulated (blue) genes among PC, SEC, and AC intestinal populations obtained from the scRNA-seq analysis of IECs in mice fed inulin diet.

**Figure S2**

**Fig. S2| Bacterial colonization in ABX-treated mice and gnotobiotic mice fed with inulin or control diet.** (A) Bacterial load measurement before and after diet treatment with or without ABX. (B) Representative histograms showing MFI in intestinal epithelial cells from mice fed an inulin diet, control diet, or inulin diet combined with antibiotic (ABX) treatment. (C) Kinetics of bacterial colonization in gnotobiotic mice monocolonized with *Bacteroides ovatus* (BOV) or *Bacteroides thetaiotaomicron* (BT) (n=6). Data were analyzed using Two-way ANOVA followed by Tukey’s multiple comparisons.

**Figure S3**

**Fig. S3| Phenotypic characterization of HIF-1α^∆IEC^ mice.** (A) Relative expression of *Hif1a* in IECs from HIF-1α^fl/fl^ and HIF-1α^∆IEC^ mice fed with inulin diet (n=4). (B) MFI for HIF-1α^+^ labeling in CD45^-^/EpCAM^+^ cells, as determined by flow cytometry, in IECs from HIF-1α^fl/fl^ and HIF-1α^∆IEC^ mice. C) Representative histograms showing the MFI peak of HIF-1α in intestinal epithelial cells from from HIF-1α^fl/fl^ mice compared to MFI from HIF-1α^∆IEC^ mice. (D) Body weight (g) of mice fed with inulin diet for 4 weeks (n=8-9). (E) Small intestine length normalized by body weight (n=8-9). (F) Cecum length normalized by body weight (n=8-9). (G) Percentage of Ki67 positive cells in EpCAM^+^/ CD45^-^ cells in the colon of mice fed with inulin diet (n=4). (H) EdU-positive cells in colon from HIF-1α^fl/fl^ and HIF-1α^∆IEC^ mice fed with inulin or control diet (n=3-4). (I) Representative images of EdU-positive cells in the colon of mice treated with inulin or control diet (n=3-4). Bars = 200 µm, 10 x magnification. (J) Crypt length of colon from HIF-1α^fl/fl^ and HIF-1α^∆IEC^ mice fed with inulin or control diet (n=3-4). Data were analyzed using Student’s t-test (A, B, D, E, F and G) or Two-way ANOVA followed by Sidak’s multiple comparisons test (H and J). In all graphs, each point represents an individual mouse. **p* < 0.05, ***p* < 0.01, ****p* < 0.001.

**Figure S4**

**Fig. S4| Epithelial deletion of HIF-1α cause minor changes in the gut microbiota.** (A) Heatmap displaying the relative abundance of bacterial phyla in the colon microbiota of HIF-1α^fl/fl^ (WT) and HIF-1α^ΔIEC^ (KO) (n=8), based on 16S rRNA gene sequencing (B) Alpha diversity of the HIF-1α^fl/fl^ and HIF-1α^ΔIEC^ colon microbiota. (C) Principal Coordinates Analysis (PCoA) based on Bray-Curtis’s dissimilarity showing the beta diversity of the HIF-1α^fl/fl^ and HIF-1α^ΔIEC^ colon microbiota (n=8). (D) LEfSe analysis showing bacterial taxa with significantly different relative abundances, based on LDA scores (n=8).

**Figure S5**

**Fig. S5| Volcano plot presenting the differentially regulated proteins in intestinal epithelial cells of HIF-1α** **^∆IEC^ and their controls on inulin diet.**

**Figure S6**

**Fig. S6| Epithelial deletion of VHL leads to a reduction in proliferating intestinal cells and on ISCs activity.** (A) Experimental design illustrating diets treatments and analyses performed in VHL^fl/fl^ and VHL^ΔIEC^ mice. (B) Mice body weight (g) (n=6-7). (C) Colon length normalized by body weight (n=6-7). (D) Crypts length (n=6-7). (E) Quantification of EdU-positive cells per crypt (n=6-7). (F) Quantification of the clonogenicity capacity of colon crypts in VHL^ΔIEC^ mice and their controls (n=6). (G) Average of organoids volume (n=5). Data were analyzed using Student’s *t*-test. In all graphs, each point represents an individual mouse. **p* < 0.05.

**Figure S7**

**Fig. S7| Epithelial deletion of HIF-1α does not affect mitochondrial mass in colon organoids**. (A) Gating strategy used in the experiment to determine mitochondrial mass in epithelial cells from colon organoids. Viable cells (negative for Live dead staining) were evaluated for EdU and MitoGreen labeling. (B) Mean fluorescence intensity (MFI) of live cells labeled with MitoGreen collected from organoids of HIF-1α^fl/fl^ and HIF-1α^ΔIEC^ treated with Etomoxir (n=4). (C) Mean fluorescence intensity (MFI) in EdU-positive or negative cells labeled with MitoGreen collected from organoids of HIF-1α^fl/fl^ and HIF-1α^ΔIEC^ treated with Etomoxir (n=4). (D) Histogram representation MitoGreen MFI of cells from organoids of HIF-1α^fl/fl^ and HIF-1α^ΔIEC^ treated with Etomoxir. (E) Percentage of EdU-positive cells in organoids from HIF-1α^fl/fl^ and HIF-1α^ΔIEC^ mice treated with oligomycin, antimycin, rotenone or 2-DG (n=3-5). Data were analyzed using Two-way ANOVA followed by Sidak’s multiple comparisons test. In all graphs, each point represents an individual mouse. **p* < 0.05, ***p* < 0.01, ****p* < 0.001.
