# Supplementary figures and images for: HIF-1 attenuates high-fiber diet-mediated proliferation and stemness of colonic epithelium
Source: Gut Microbes. 2025 Aug 19;17(1):2543123. doi: 10.1080/19490976.2025.2543123 (PMC12369635; doi:10.1080/19490976.2025.2543123)

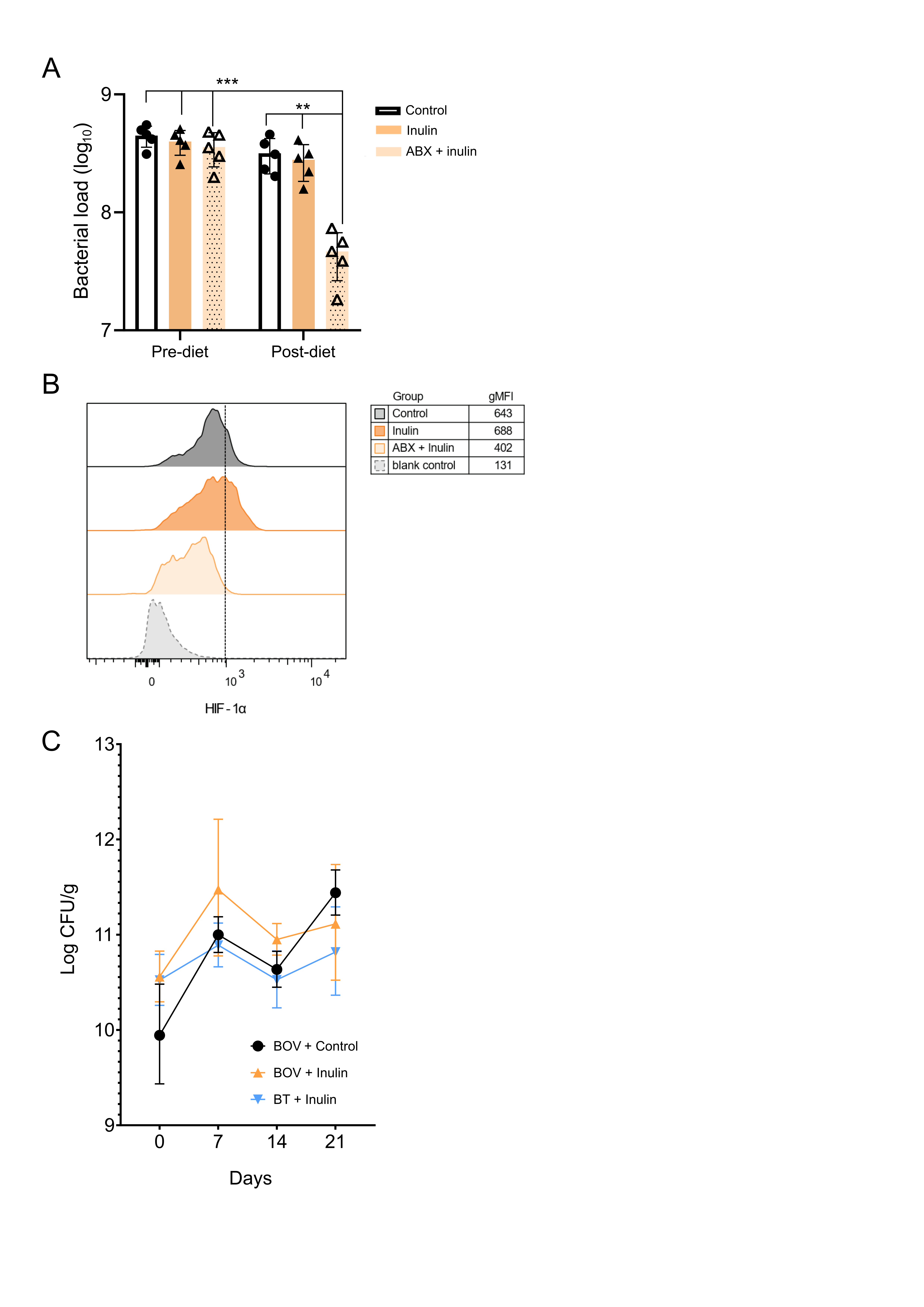

Supplement: Supplemental Material [file KGMI_A_2543123_SM5400.zip › kgmi-s-2025-0298-20250731041635/graphic/S2_compressed.tiff]

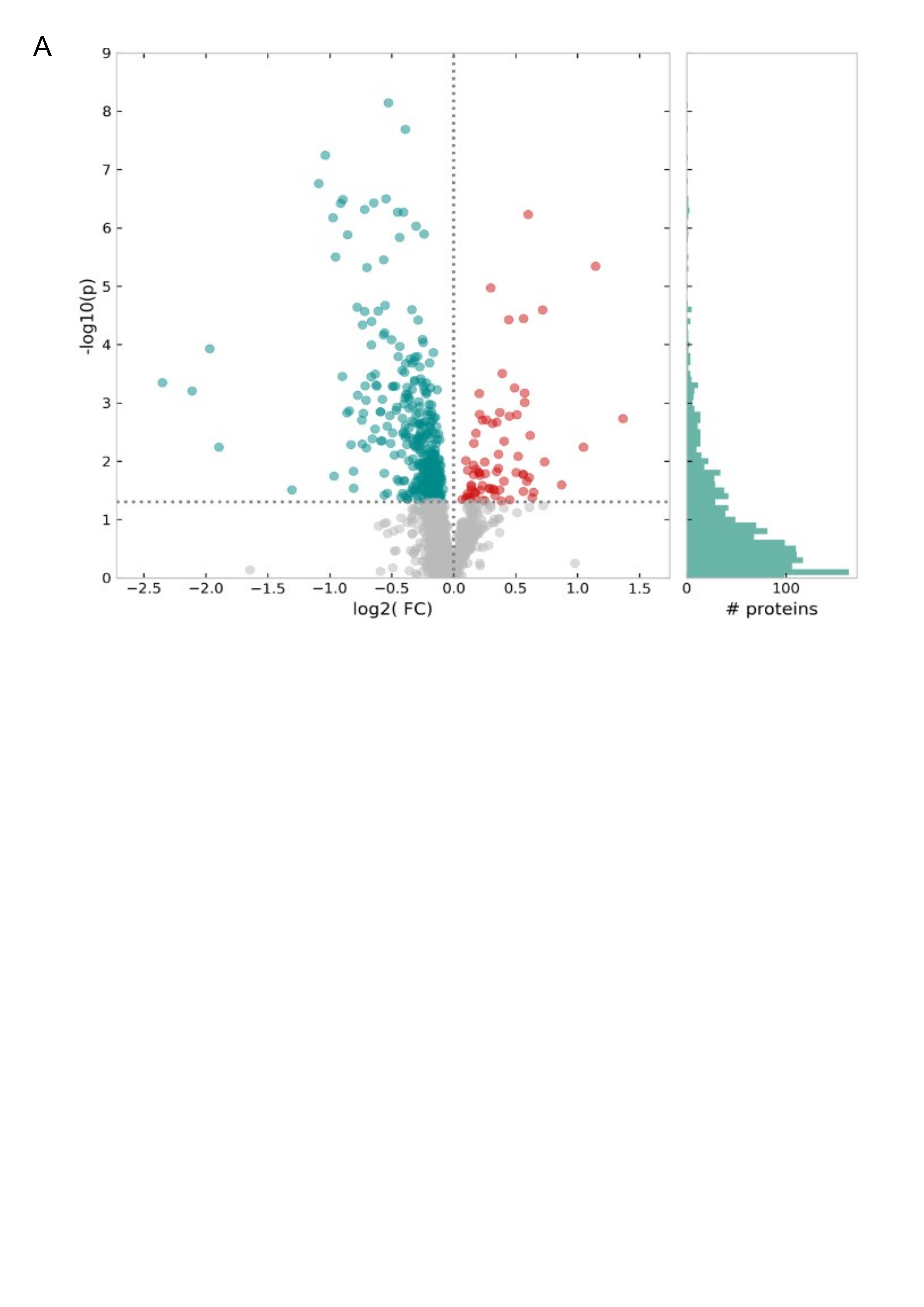

Supplement: Supplemental Material [file KGMI_A_2543123_SM5400.zip › kgmi-s-2025-0298-20250731041635/graphic/S5a_compressed.tiff]

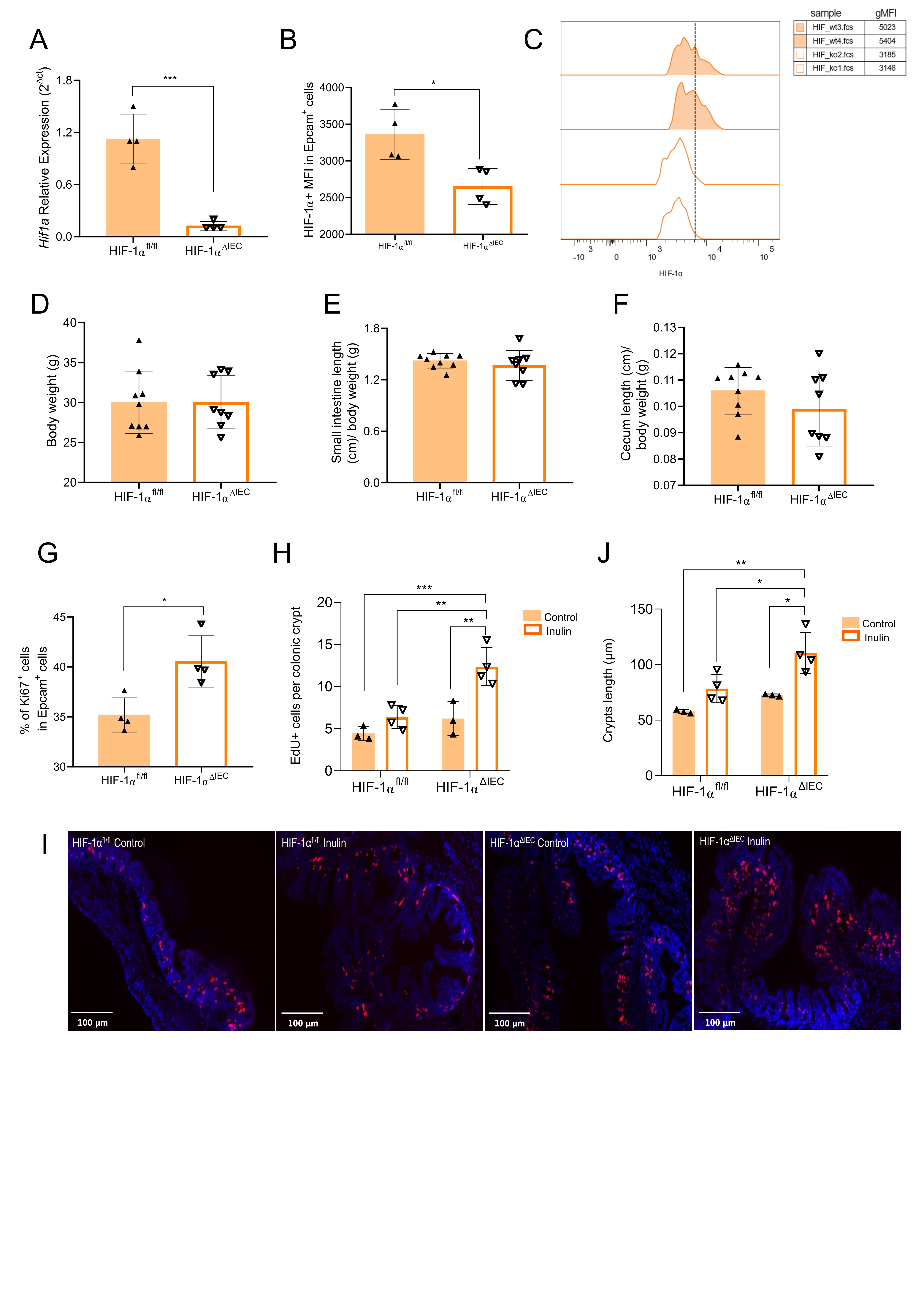

Supplement: Supplemental Material [file KGMI_A_2543123_SM5400.zip › kgmi-s-2025-0298-20250731041635/graphic/S3_compressed.tiff]

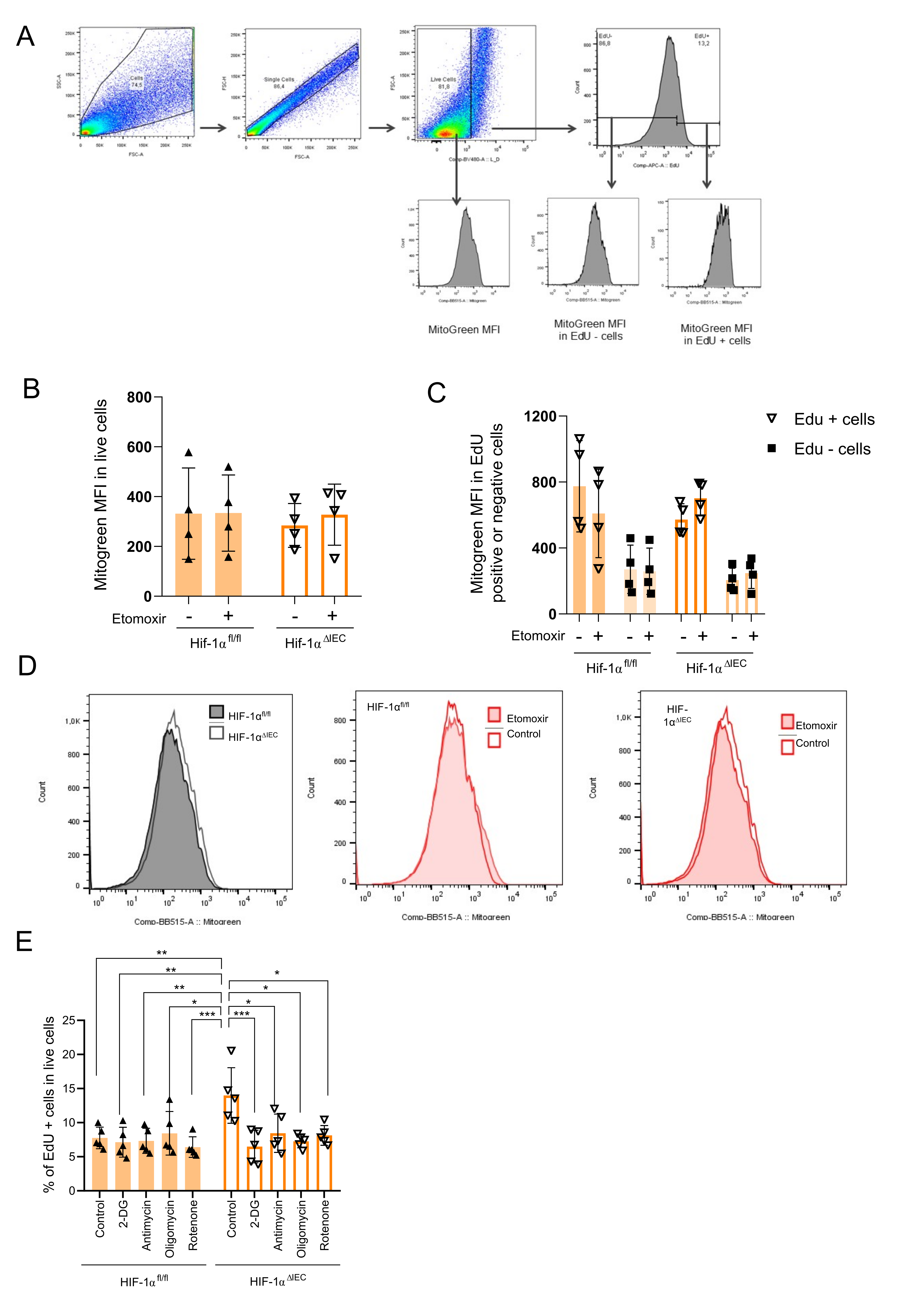

Supplement: Supplemental Material [file KGMI_A_2543123_SM5400.zip › kgmi-s-2025-0298-20250731041635/graphic/S7_compressed.tiff]

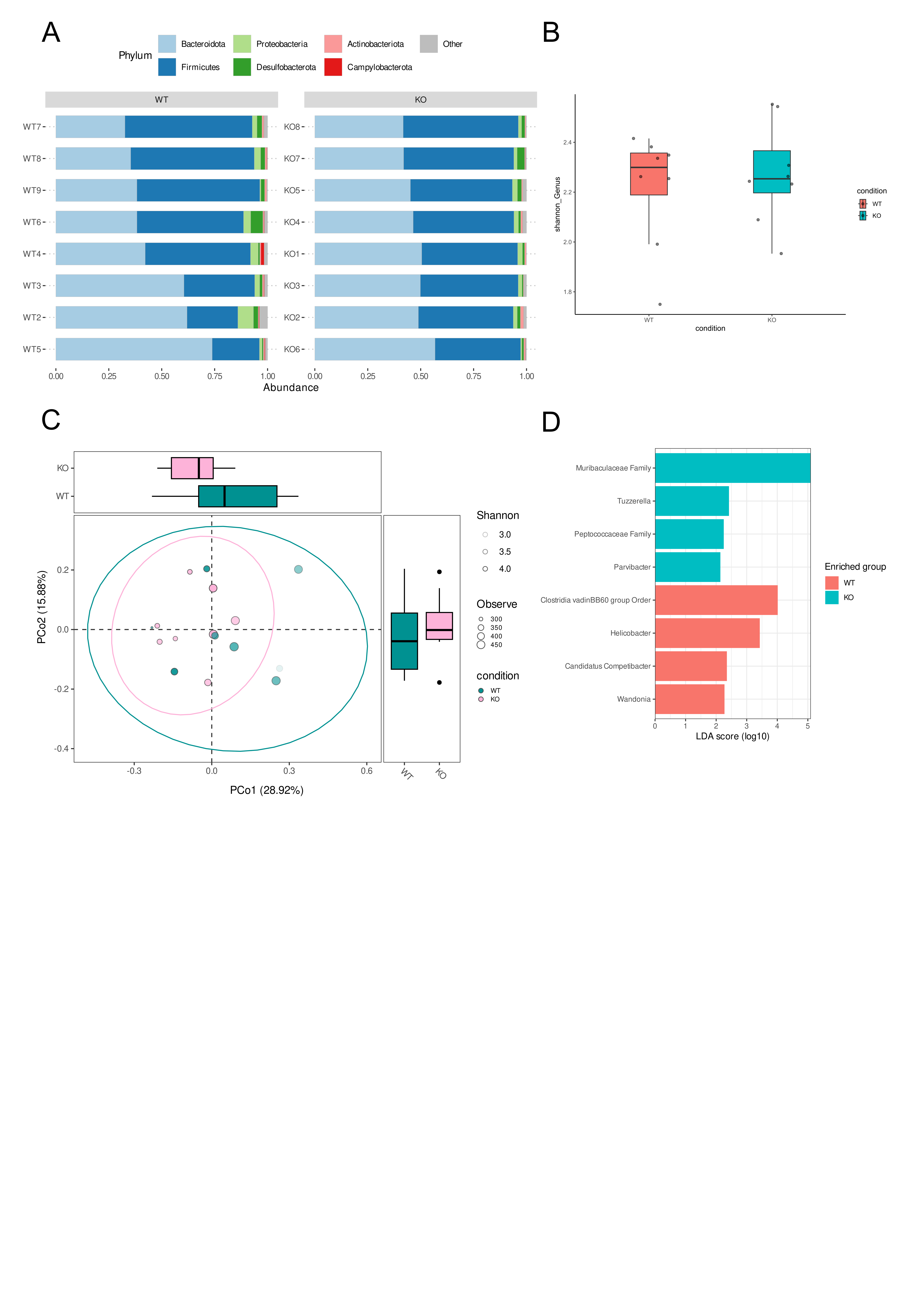

Supplement: Supplemental Material [file KGMI_A_2543123_SM5400.zip › kgmi-s-2025-0298-20250731041635/graphic/S4a_compressed.tiff]

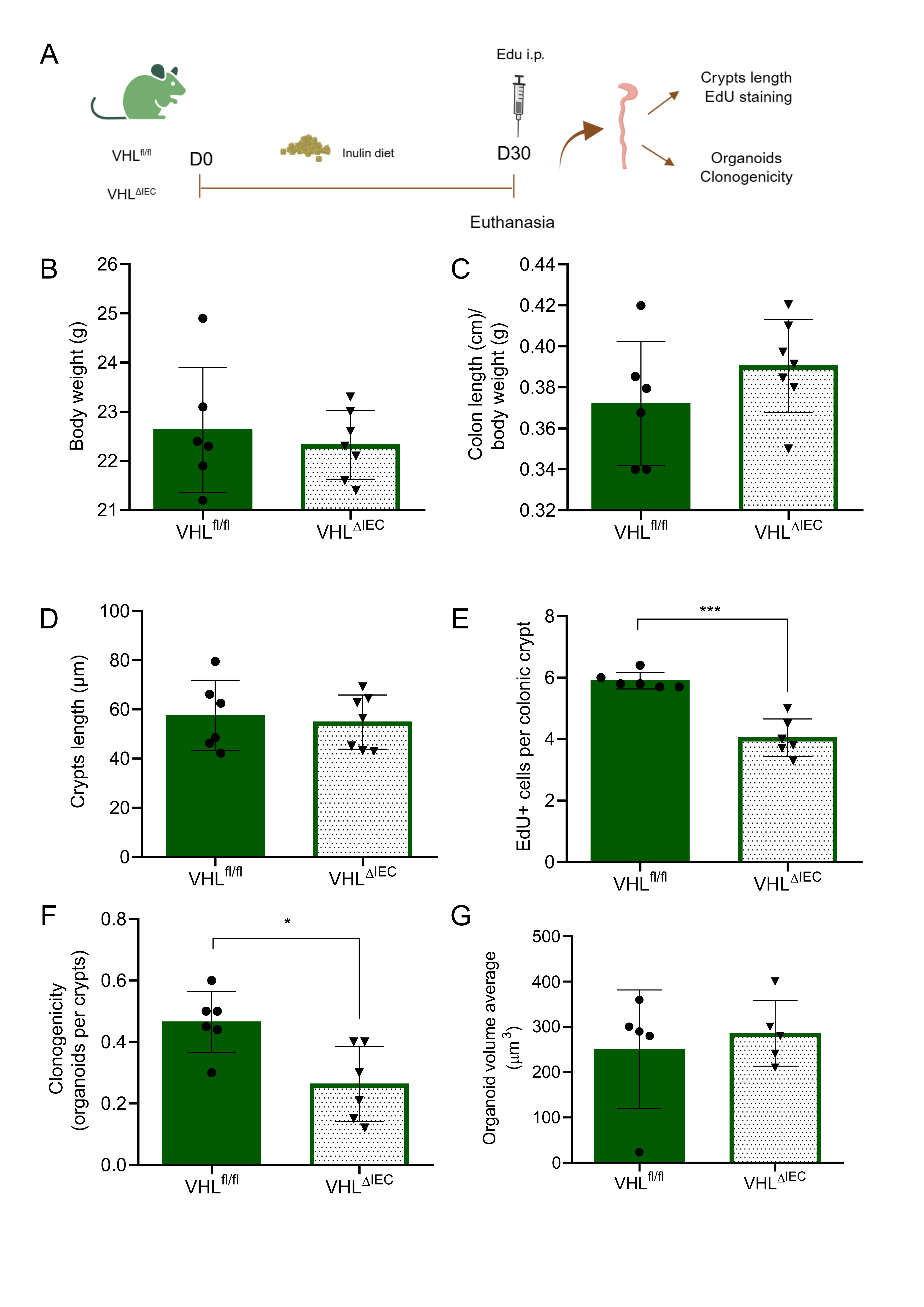

Supplement: Supplemental Material [file KGMI_A_2543123_SM5400.zip › kgmi-s-2025-0298-20250731041635/graphic/S6_compressed.tiff]

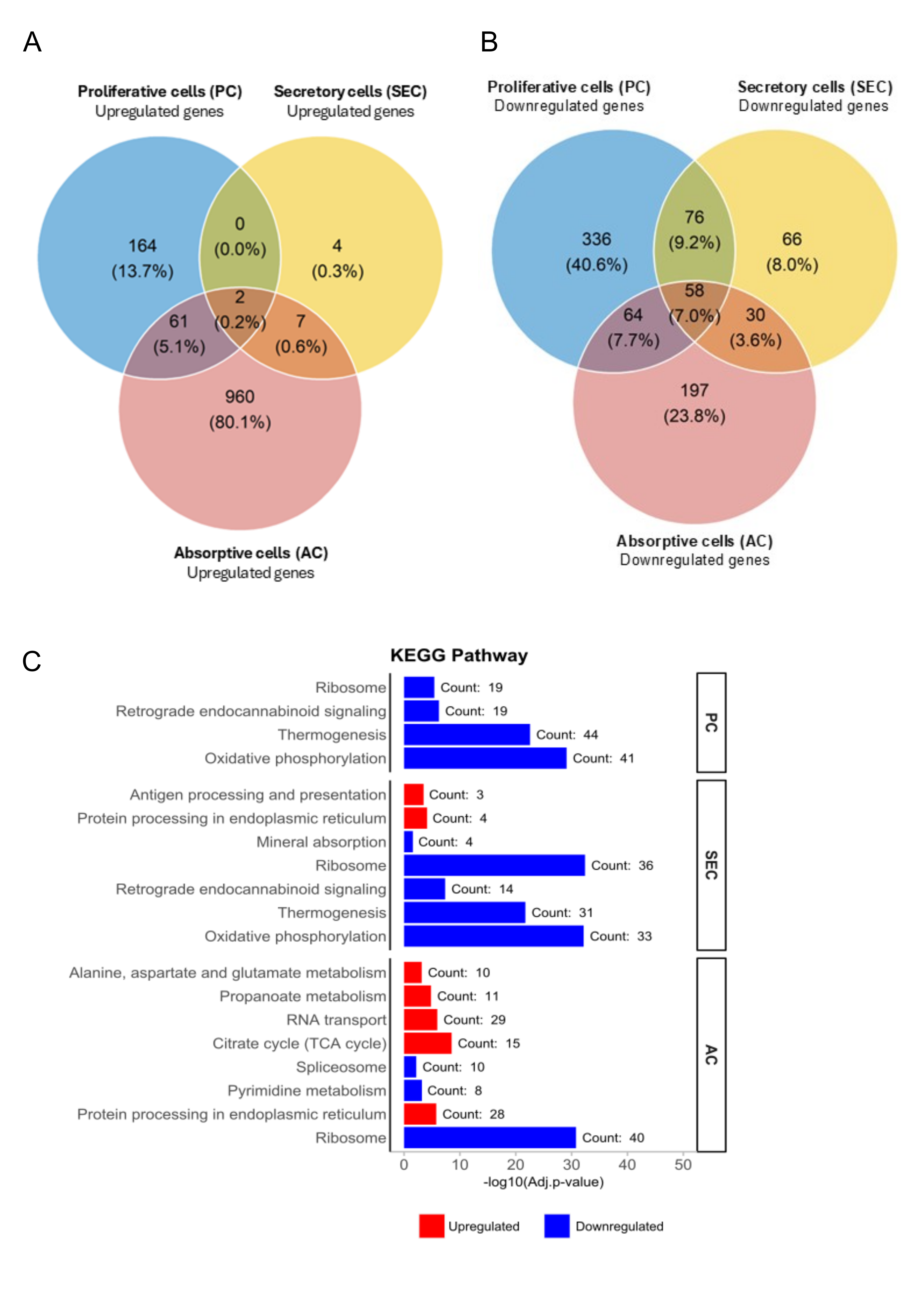

Supplement: Supplemental Material [file KGMI_A_2543123_SM5400.zip › kgmi-s-2025-0298-20250731041635/graphic/S1_compressed.tiff]
